# A quasi-experimental study in sibling dyads: differential provocation-aggression patterns in the interactive taylor aggression paradigm

**DOI:** 10.3389/fpsyg.2024.1288743

**Published:** 2024-02-08

**Authors:** Julia Koch, Lucia Hernandez-Pena, Charlotte Keeler, Edward S. Brodkin, Ute Habel, Rik Sijben, Lisa Wagels

**Affiliations:** ^1^Department of Psychiatry, Psychotherapy and Psychosomatics, Faculty of Medicine, RWTH Aachen, Aachen, Germany; ^2^JARA – Translational Brain Medicine, Aachen, Germany; ^3^Department of Psychiatry, Perelman School of Medicine at the University of Pennsylvania, Philadelphia, PA, United States; ^4^Research Center Jülich, Institute of Neuroscience and Medicine: JARA-Institute Brain Structure Function Relationship (INM 10), Jülich, Germany; ^5^Brain Imaging Facility, Interdisciplinary Center for Clinical Research (IZKF), RWTH Aachen University, Aachen, Germany

**Keywords:** taylor aggression paradigm, reactive aggression, competition, social interaction, sibling dyads

## Abstract

**Introduction:**

The Taylor Aggression Paradigm (TAP) is a well-established tool for assessing provocation-induced reactive aggression. We introduce an interactive version, the iTAP, with real-time opponents across 60 trials, including five simulated provocation trials in the middle. In this quasi-experimental study, we evaluate the effectiveness of the paradigm to investigate reactive aggression in interacting participants. The design allows us to employ the TAP in settings of high familiarity dyads, addressing an existing gap.

**Method:**

Twenty-eight healthy same-sex adult sibling pairs (*N* = 56) competed against each other in the iTAP, exemplifying high familiarity through their social and emotional co-development, and mutual knowledge. Additionally, we explore naturally arising aggression types in terms of sibling pairs’ reciprocal aggression trajectories across trials. Lastly, we investigate situational and personal variables influencing reactive aggression on the iTAP within high familiarity dyads.

**Results:**

In line with non-interactive TAP versions, siblings employed a global “tit-for-tat” strategy in response to heightened provocation: Aggression increased during manipulated trials of increasing provocation, persisted during real interaction and declined in the final block, suggesting sibling co-regulation which was underscored by the convergence in within-pair aggression level. We found no gender differences in these dynamics but a trend for higher initial aggression levels within brother pairs and higher responsiveness to increased provocation in sister pairs. Overall aggression levels were related to situational variables including trial outcome (lost, won, and tie), Further, siblings’ state anger correlated positively with aggression scores on the iTAP. Aggression was not reliably related to personal variables predicting aggression. We identified subgroups of sibling pairs with distinct provocation-aggression patterns related to differences in reported behavioral motivations and emotional states. The results highlight situational over personal variables in determining aggressive behavior on the task in this sample of healthy adults. While no direct link between sibling relationship quality and aggression was found, the overall behavior was likely influenced by the familiarity between siblings and the specific context of their relationship.

**Conclusion:**

The iTAP demonstrates promise as a tool for studying reciprocal aggressive behavior. The emergence of different interaction patterns underscores the ecological validity introduced by the interactive context, which complements the standard versions of the TAP.

## 1 Introduction

In social interactions, people may decide to collaborate or compete to reach a goal. This social decision is strongly influenced by the specific type of interaction and the actors involved. For example, a situation perceived as competitive can quickly become hostile and lead to aggressive behavior. Additionally, one-sided provocations can trigger aggressive reactions in the other party. This type of aggression is known as reactive aggression, as distinct from aggression used as a strategic tool, termed proactive aggression (Dodge, 1991). Reactive aggression is defined as a behavior often triggered by perceived threat, provocation, or frustration with the intent to harm others (Berkowitz, 1993; Allen et al., 2018). At a given moment, the likelihood of reactive aggressive behavior to emerge is shaped by a combination of situational variables, such as external provocation, and personal variables including emotion, cognition, and arousal, a concept that is formulated within the General Aggression Model (GAM; Anderson and Bushman, 2002). These factors influence internal states, forming a bottom-up mechanism in the emergence of aggressive behavior, while the behavioral consequences impact future actions.

Naturalistic observation studies (Dickson et al., 2015) are limited by their inability to induce controlled conflict or specific provocations, making it difficult to systematically study the impact of situational factors. However, laboratory studies may not accurately reflect real-world social dynamics (Giancola and Parrott, 2008; Muller et al., 2012) and, while providing control over experimental conditions and the range of provocations, they lack the naturalness and authenticity of real interactions. To systematically study the factors that lead to aggression, so far various versions of laboratory reactive aggression paradigms have been applied. The Taylor Aggression Paradigm is among the most established paradigms for studying reactive aggression in competitive settings (TAP; Taylor, 1967). Participants play multiple rounds against a perceived but ostensible opponent, with both players selecting the level of punishment for the opponent at the start of each trial. The winner of a round determines the punishment that is deduced from the loser of the round. The TAP has been adapted to incorporate various punishment modalities, such as aversive auditory stimulation (Chester and Lasko, 2018), mechanical stimulation (Veit et al., 2010), thermal stimulation (Krämer et al., 2011) and monetary punishments (Wagels et al., 2018; Weidler et al., 2019). These studies have used manipulated punishment levels to examine how different levels or intensities of provocation influence aggressive behavior. However, they typically use preprogrammed opponents with random provocation intensities (Wagels et al., 2018; Weidler et al., 2019; Konzok et al., 2020) or grouped into predetermined provocation blocks.

This research aims to fill in the gap in the literature by introducing an interactive version of the TAP (iTAP) to investigate reciprocal dynamics of aggression in dyadic interactions in a systematic yet ecologically valid manner. In our iTAP, we use monetary provocation as punishment, which is in line with previous TAP versions (e.g., Wagels et al., 2018). Anderson et al. (2008) previously employed a dyadic version of the TAP to explore reciprocal aggression in college student dyads, but to the best of our knowledge, real interactions within the TAP have not been studied in dyads with close relationships. There are prior studies which use the TAP or its derivatives on dyads from close relationships (e.g., romantic partners or friends: Chester et al., 2021; twins: Dinić et al., 2020). Familiarity has a strong influence on aggressive behavior, with interactions involving more familiar individuals often leading to more frequent or more easily provoked aggressive behavior compared to interactions with strangers (Hessick, 2007). In these settings, expectations and anticipated consequences of actions are closely linked to individuals’ knowledge of each other’s past behaviors. This familiarity gives individuals the ability to predict their interaction partner’s reactions, enabling them to make informed decisions and adjust their actions accordingly. Recreating such a dyad-specific condition within preprogrammed provocation settings is challenging.

Therefore, the purpose of this research is to explore reactive aggression in the TAP during real interactions among highly familiar individuals, particularly adult siblings. Although previous studies have investigated interactions within competitive settings in close relationships, non-simulated interaction within this specific relationship remains underexplored.

### 1.1 Sibling context and aggression-mediating factors

Sibling relationships are a unique type of familiarity context, as social dynamics are usually acquired and maintained over time within the sibling bond (Brody, 2004). Despite siblings being highly suitable, they have been an underrepresented sample population in aggression research (Hicks et al., 2013). Studying siblings is particularly interesting due to their co-development of aggressive behavior and competitive attitudes among siblings. It has been shown that sibling relationships have a significant impact on individuals’ developmental, psychological, and behavioral outcomes (Hudson and Trillmich, 2008). In explicit, evidence suggests a reciprocal influence between siblings in the development of aggressive behaviors (Williams et al., 2007; Natsuaki et al., 2009). Moreover, the sibling relationship offers a safe environment for learning and experimenting with aggression, as it generally does not pose a substantial threat to the persistency of the relationship (Campione-Barr et al., 2018).

According to the social comparison theory, people compare themselves to those they perceive as similar and/or close to them (Garcia et al., 2013). Therefore, siblings, mostly having a shared early environment and mutual experiences, are highly prone to compare and compete against each other. In line with this, same sex and closeness in age between siblings are contributing factors to sibling rivalry (Salmon and Hehman, 2015). Although the relationship between siblings becomes more harmonious with age, it was shown that siblings still compete in areas such as socioeconomic status and general success in life (Sumijati and Widhi, 2018). Additionally, sibling rivalry established during childhood can have a prolonged effect on externalization problems, including aggressive behavior (Stocker et al., 2002). In contrast, siblings also hold the potential to cooperate as suggested by Hamilton’s kin selection theory (Hamilton, 1964; Neyer and Lang, 2003), which outlines, that individuals may engage in more altruistic or cooperative behaviors toward their relatives because they share a proportion of their genes. Assessing dyadic interaction within the iTAP allows us to investigate the interplay between situational factors, such as provocation level or relationship attitudes, and personal factors, such as personality traits.

#### 1.1.1 Situational factors related to conflict in the sibling relationship

When looking into aggression and provocation between siblings, factors such as the sex of the siblings play an important role. Same-sex sibling pairs have been associated with more stable levels of intimacy, with pairs of sisters perceiving the sibling relationship as more positive than pairs of brothers (Kim et al., 2006). Additionally, brothers are generally more likely to compare themselves to their sibling and react more strongly to unequal parental treatment than sisters (Buist et al., 2013). In general, being male has been identified as a risk factor for aggressive behavior (Tevlin, 2021), particularly in terms of physical aggression (Archer, 2004).

In the context of sibling relationships, dominance traits have been linked to rivalry and competition between siblings (Sulloway, 2010). Birth order can be a contributing factor in determining sibling dominance, with older siblings often showing more dominance over younger siblings (Tucker et al., 2013; Lindell and Campione-Barr, 2017). Nonetheless, it has previously been found that in adolescence, dominance behavior in the younger sibling contributes to sibling conflict (Tucker et al., 2010). Independent of age order, siblings with high-dominance traits displayed more competitive behavior and a relatively apathetic sibling relationship during an interactive version of the Chicken Game task. Siblings with low-dominance traits showed a more affectionate and reciprocal relationship and cooperative turn-taking strategies (Hernandez-Pena et al., 2023).

#### 1.1.2 Personal factors associated with reactive aggression

Anger has been identified as a significant factor in the emergence of aggression (Blair, 2018). State anger leads to aggressive behavior under more serious external influences, whereas trait anger predicts aggressive responding even under more minor provocations (Richard et al., 2023). Furthermore, the link between anger (Quan et al., 2022), trait aggressiveness (Bettencourt et al., 2006; Wilkowski and Robinson, 2008) and aggressive behavior is likely mediated by hostility. Impulsivity (Gómez-Leal et al., 2022; Meidenbauer et al., 2023), and self-control (Vazsonyi et al., 2017) have also been associated with aggressive behavior. Further, trait aggression has been associated with reactive aggression in the TAP (Krämer et al., 2008; Repple et al., 2017; Weidler et al., 2019). In a repeated dictator game, dominance motive has been found to predict social decisions with higher dominance motivated individuals tending to engage in more retaliatory behavior (Suessenbach et al., 2019).

### 1.2 Objectives

The primary objective is to assess the effectiveness of the iTAP as a tool for studying bidirectional provocation-induced aggression in sibling relationships. Therefore, we examine how aggression unfolds across consecutive task blocks, including the presence of a block with manipulated provocation levels. In contrast to Anderson et al. (2008), we introduce five manipulated trials (a fake block) with increasing provocation levels in addition to actual interactive trials allowing the comparison of real and manipulated trials and studying the aggression regulation process. Additionally, we aim to investigate the correlation between the degree in overall aggression displayed in the iTAP and personal variables previously linked to aggressive behavior. Our secondary objective is to understand the dynamics of dyadic aggression during the iTAP and to identify naturally occurring sibling types in terms of aggression patterns. For this purpose, we combine dyadic scores on a trial-by-trial basis to capture the reciprocal nature of aggression and aim to identify different clusters of dyads defined by their unique aggression trajectories. Our third objective is to describe these identified sibling aggression types on situational variables (e.g., sibling relationship quality, provocation) and personal variables (e.g., traits and personality factors, sex), as well as task ratings (e.g., motivation, emotional state, evaluation of other and strategies) while considering both the dyadic total expression and sibling differences.

### 1.3 Hypotheses

In line with previous research (Anderson et al., 2008; Weidler et al., 2019; Dinić et al., 2020; Konzok et al., 2020), we anticipate that the mean level of aggression among sibling pairs will increase across trials and blocks, with the highest levels of aggression occurring after the fake block in the center. Building on prior findings (Weidler et al., 2019), we hypothesize that gender will influence overall aggression and distinctly modulate the aggression trajectory across blocks differently. We anticipate that brothers display higher levels of aggression, whereas sisters may be more responsive to provocation (Bettencourt and Miller, 1996). Additionally, we expect a positive correlation between mean individual aggression and psychometric constructs (Blair, 2018; Weidler et al., 2019; Meidenbauer et al., 2023) as well as measures of attenuated sibling relationship quality (Hernandez-Pena et al., 2023).

We anticipate that subgroups of siblings will employ either a mutual consent strategy ("Fairness strategy"), characterized by a shared level of aggression, or a divergent strategy ("Competitive strategy"), characterized by trial-based differences in aggression levels between members, reflecting siblings’ cooperative and competitive nature (Hamilton, 1964; Neyer and Lang, 2003; Salmon and Hehman, 2015). We expect that clusters will differ in sibling relationship ratings as well as overall traits related to aggression and dominance. For example, clusters with lower aggression levels may have higher mean positive sibling relationship scores, while clusters with higher aggression levels may have higher levels of sibling conflict and rivalry. Further, we expect that sibling clusters using a tit-for-tat strategy and potential revenge-related behavior are likely to have higher levels of overall dominance traits or a significant difference in this trait between members (Tucker et al., 2010; Suessenbach et al., 2019).

## 2 Materials and methods

### 2.1 Open science and ethical preconditions

The study was preregistered including study design, planned sample size, inclusion and exclusion criteria, and planned primary analyses (accessible at).^1^ In line with the preregistered plan, we conducted a comparison of aggression scores across blocks, while introducing sibling gender as a factor. The planned analysis of the actor-partner interdependence model was not incorporated within this paper and will be published elsewhere. Similarly, hormonal data were omitted due to quality concerns and the requirement for extensive subject exclusion. Additionally, we introduced a cluster analysis and subsequent comparisons between clusters, as analyses not initially outlined in the preregistration.

The study was approved by the internal ethics committee. All participants submitted informed written consent in accordance with the Declaration of Helsinki (World Medical Association, 2013) before participation and were compensated with 30 euros each.

### 2.2 Participants

Data collection for this study took place between May 2021 to September 2022 at the University Hospital RWTH Aachen, Germany. Participants were recruited via locally distributed flyers, student platforms, and social media. Interested candidates completed an online screening survey using Google Forms. To meet the inclusion criteria, candidate pairs had to be siblings of the same sex (biologically defined based on siblings’ self-reports), have a maximum age difference of 5 years, and be full siblings who co-resided at least 10 years. All included participants were between 18 and 35 years old, fluent in German, and had no history of psychiatric or neurological disorders.

To ensure compliance with the last criterion, participants underwent a screening process using a short version of the Structured Clinical Interview for DSM-IV (SCID IV) for Axis I disorders (Wittchen et al., 1997). If necessary, participants were further interviewed by a psychologist to confirm their eligibility.

As part of the broader study, saliva samples were collected from participants. To ensure the salvia sample quality for hormonal assessments, participants who were smokers were excluded from the study. In addition, all sibling pairs were scheduled to arrive between 4 and 6 pm to account for natural hormone fluctuations throughout the day. Hormonal measurements were not included in this analysis due to quality control issues with the saliva samples of 8 pairs. The final sample for this study consisted of 28 sibling pairs, with 13 pairs being male, and a mean age of 23.39 years (ranging from 18 to 33, *SD* = 3.42) and mean age difference between siblings of 2.3 years (*SD* = 1.41).

### 2.3 Interactive taylor aggression paradigm (iTAP)

**FIGURE 1:**
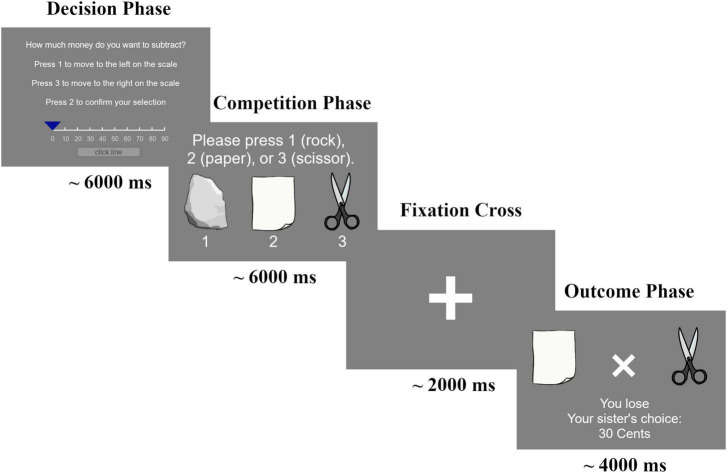
Example trial of the iTAP. The trial consisted of three phases: In the *Decision Phase*, participants had 6 s for their monetary selection by pressing a button on the keyboard. In the *Competition Phase*, participants chose one of the three game options (rock, paper, or scissors) displayed on their monitor within a 6-s window. In the *Outcome Phase*, participants saw one of three possible game outcomes (win, loss, or tie) as well as their sibling’s choice in monetary punishment displayed on the monitor.

In the standard TAP, participants engage in repeated rounds of reaction time games against a simulated opponent. In our interactive Taylor Aggression Paradigm (iTAP, see Figure 1) version, siblings engaged in dyadic interactions on the task, following a quasi-experimental design. We replaced the reaction time game with a rock-paper-scissors game and introduced a real player as opponent, the sibling. By incorporating the rock-paper-scissors game, we aimed to enhance the ecological validity of the paradigm by minimizing potential confounds arising from participant suspicions regarding the manipulation of the reaction time game.

Each iTAP trial began with both players selecting the amount of monetary punishment (ranging from 0 to 90 cents in increments of 10) that was deducted from the other player in the event of the other player’s loss. Failure to respond in this phase resulted in a fixed deduction of 100 cents from the non-responder to motivate participants to respond. Consequently, in such cases, the opponent was guaranteed to win the trial, regardless of the game outcome. Following the competition phase, the opponent’s punishment selection was revealed to each participant, along with the outcome of the game. The winner received a fixed monetary gain of 50 cents and the loser lost the amount of cents the other player selected at the trial start. In subsequent rounds, each player had the opportunity to react to the opponent’s decision.

Unknown to the participants, the outcome of the rock-paper-scissors game was manipulated, and they were exposed to a win, loss, or tie trial (in equal proportions and with a fixed order across dyads) with the outcome being opposing for both players, except in the tie trials. The monetary punishment selection displayed was the actual selection of the other player, except for the first five trials in the third block (fake block). In the fake block, participants observed an increasing punishment selection by the opponent (30-50-50-60-70 cents). This block was designed to generate conflict between the players and introduced provocation, particularly for pairs who had chosen a non- or low-punishment strategy (e.g., selecting 0 cents) until that point. Overall, participants engaged in four blocks of 15 trials each, along with a continuous fake block of 5 trials preceding the third block. The task was implemented using the experimentation software PsychoPy Version 2020.2.10 (Peirce et al., 2019).

### 2.4 Procedure

The sample took part in a broader study including a total of three tasks followed by a set of self-report instruments, taking 2.5 h in total. Participants also played two other interactive tasks, including a Tetris Cooperative Task and an interactive version of the Chicken Game task (see Hernandez-Pena et al., 2023 for details). Before each task participants received instructions through a pre-recorded presentation to ensure consistency in explanation. They were informed that they could win up to 10 euros as an additional incentive in the tasks, depending on their scores, to motivate a competitive or cooperative attitude. Due to manipulation of the game outcomes in the iTAP, it was not feasible to reward participants based on their individual game performance. As a result, all participants received the maximum amount of money at the end of the study. Siblings were explicitly instructed not to communicate about the tasks after instructions to prevent potential arrangements beforehand.

During the tasks, participants were seated in two adjacent lab rooms in front of a monitor. The virtual server was operated from outside the lab rooms (for more information see Supplementary materials “Server Communication”). The three tasks were completed in a fixed order for the entire sample, starting with the Cooperative Tetris Task, followed by the interactive Chicken Game task and finishing with the iTAP. At the end of each task block, participants were asked to answer several questions related to their personal motivation, opinion on the outcome and fairness, as well as emotional states after winning or losing trials (see Supplementary Table 1). At the end of each task, participants answered additional questions about their own and their sibling’s intentions and strategies during the task. Additionally, hormonal samples (cortisol and testosterone) were collected at four time points: one baseline sample at the start of the experiment and one after each task.

After finishing all three tasks, participants completed German versions of various self-report instruments accessed through the online platform SoSci Survey. At the end of the session, participants were briefly interviewed about their general task strategies. Regarding the iTAP task, participants were also asked whether they suspected any manipulation of the rock-paper-scissors game outcomes or provocation manipulation during the fake trials. None of the participants suspected any manipulation of game outcomes or questioned the overall authenticity of playing against their sibling.

### 2.5 Instruments and task rating scales

Sibling relationship quality was assessed with two instruments. First, the Sibling Type Questionnaire (STQ; Stewart et al., 2001) assessed the perception of participants’ relationship with their siblings across including mutuality, competition, criticism, apathy, and longing subscales. Second, the Adult Sibling Relationship Questionnaire (ASRQ; Stocker et al., 1997), assessed various aspects of adult sibling relationships. Due to technical issues with data collection on the SoSci platform some data was missing. We only included the subscales of the ASRQ with complete items namely acceptance, emotional support, intimacy, instrumental support, and knowledge. Dimensions related to aggression were assessed with the Buss-Perry Aggression Questionnaire (BPAQ; Buss and Perry, 1992), comprising four subscales: physical aggression, verbal aggression, anger, and hostility. Motives related to social hierarchies were assessed with the Dominance, Prestige and Leadership Motives Questionnaire (DoPL; Suessenbach et al., 2019). Social behavior in relation to hierarchies was assessed with the Rank Style with Peers Questionnaire (RSPQ; Zuroff et al., 2010). Different aspects of anger were measured with the State-Trait Anger Inventory 2 (STAXI-2; Spielberger, 2010), encompassing subscales the state anger, trait anger, anger expression-out, anger expression-in, anger control. Two subscales of the competitive attitude scale (CAS) focused on competition in the context of personal development and self-improvement [Personal Development Competitive Attitudes (PDCA) Scale (Ryckman et al., 1996)] and extreme attitudes (Hypercompetitive Attitudes (HCA) Scale (Ryckman et al., 1990)). Sibling’s subjective sense of power in social situations was accessed with the Sense of Power Scale (SoPS; Anderson et al., 2012). The Machiavellianism Scale (Mach IV; Christie and Geis, 1970) measures the personality trait of Machiavellianism, which is characterized by manipulative behavior. Again, technical issues with SoSci survey led to discarding of several items. Impulsiveness was assessed with the Barratt Impulsiveness Scale 11 (BIS-11; Patton et al., 1995), with subscales measuring attentional, motor, non-planning impulsiveness. Lastly, the NEO Five-Factor Inventory (NEO-FFI IV; McCrae and Costa, 2004) was administered, which captures five broad dimensions of personality including openness to experience, conscientiousness, extraversion, agreeableness, and neuroticism.

The STQ was administered online as part of the screening survey via Google forms, while all other instruments were provided onsite via SoSci Survey (Leiner, 2019; all other), accessible at^2^.

All instruments, except for the BIS and the NEO-FFI IV, were considered for analyses in the study. In addition, task rating scales were created and administered between task blocks and at the end of the task to the siblings. The ratings assessed participants’ strategies on the tasks, appraisal of fairness as well as emotional states (see Supplementary Table 1).

### 2.6 Data analysis

Data was prepared with Matlab version R2017b (The MathWorks, Inc, 2017) and checked for normality using the Shapiro-Wilk test. We identified potential outliers through boxplots using the interquartile range method. However, given the small size of our study sample, distinguishing between outliers and natural variation is challenging and may lead to biased results. Consequently, we opted not to remove outliers. We employed non-parametric statistical tests due to violations of normality of the task aggression scores, such as Spearman correlation, which are less sensitive to the influence of outliers. Reliability analysis, using Cronbach’s alpha, was performed with SPSS version 29.0 (IBM Corp, 2022) to assess the internal consistency of all included instrument subscales.

Additional analyses were either conducted using R version 4.2.2 (R Core Team, 2022) and R Studio (Posit team, 2022), and the TraMineR package (Gabadinho et al., 2011) or geepack package (Halekoh et al., 2006), or SPSS version 29.0 (IBM Corp, 2022). Non-parametric tests were selected when the assumption of normality was violated.

### 2.7 Power analysis

Due to funding and time constraints, our sample size was a priori constrained, resulting in *N* = 28 pairs (*N* = 56 subjects). Consequently, a sensitivity power analysis was performed using G*Power 3.1. We focus on the primary analysis, the manipulation check, which validates the task by comparing dyad mean and difference aggression scores across blocks and gender. This involves two separate Generalized Estimating Equations (GEE) analyses. Analysis in G*Power was conducted as linear multiple regression with an alpha level of .05 and two predictor variables. Our sample of 28 dyads would provide 80% power to detect a moderate-to-large effect size of 0.39 (Lovakov and Agadullina, 2021). While the statistical power of this analysis may be limited for detecting small effects, the primary focus of this study is to robustly capture and generalize findings pertaining to large effect sizes, which hold significance in validating the foundation of this paradigm.

### 2.8 Data preparation

In this study, aggression was operationalized as trial-wise monetary selection.

#### 2.8.1 Primary outcome measures

*Individual Scores:* First, mean aggression scores for each sibling were calculated for each distinct block of the task, treating the fake trials as a separate block. Consequently, a total of five blocks mean aggression scores were derived. Additionally, mean aggression scores across all interactive blocks were calculated.

*Dyad Scores:* For each block, we computed both the difference and mean aggression scores of the two siblings within each pair. Total block scores were created by averaging across these dyad mean and difference aggression scores, separately for each block.

#### 2.8.2 Secondary outcome measures

*Individual Scores:* For all instruments included, individual sum scores were calculated per subscale, taking item polarity into account and using mean imputation for missing items. For all task ratings occurring after the task blocks, individual scores were averaged across blocks.

Additionally, several scores were calculated for cluster comparisons. Mean aggression scores were calculated for both the 30 trials before and the 30 trials after the fake block. Subsequently, a difference score was generated by subtracting the mean aggression after the fake block from the mean aggression before the fake block (“Pre-Post Aggression Difference”). Moreover, the trials in which siblings selected zero as the aggression score were summed across all interactive trials (“Zero Aggression Trials”) and across all fake trials (“Zero Aggression Fake Trials”), respectively.

Lastly, the aggression scores across all trials (interactive and fake trials) were grouped based on the outcome of the preceding trial (win, loss and tie) and a mean aggression score for each outcome was calculated.

*Dyad Scores*: Dyad mean and difference scores were calculated for the variables “Pre-Post Aggression Difference”, “Zero Aggression Trials”, and “Zero Aggression Fake Trials”. Further, mean and difference scores were calculated for those instrument subscales and task ratings included for cluster comparisons (see Section 2.11 “Cluster ***c***omparison: instruments and task ratings”).

A summary of all included iTAP scores can be found in the Supplementary Table 2.

### 2.9 Manipulation check and correlations

With the aim to validate the iTAP task, we performed two main analyses. First, two GEE models were set up, to investigate potential changes in the dependent variables dyadic mean and difference aggression scores across task blocks and gender. This measure is the key evaluation for assessing task effectiveness in measuring and inducing provocation-induced aggression and serves as a behavioral manipulation check to detect changes in dyadic aggression following external provocation. Second, the effect of game outcome (win, lose and tie) on subsequent aggression levels was evaluated using a Kruskal-Wallis test on the mean aggression scores preceding the three outcomes.

As aggression scores were not normally distributed, spearman correlations were calculated to examine the relationship between the *individual* mean aggression scores across all trials, all interactive trials, all fake trials, and the total scores of the subscales of all instruments included. Likewise, Spearman correlations between *dyadic* mean and difference aggression scores across all trials, all interactive trials, and all fake trials, and the total scores of all subscales included were performed.

### 2.10 Grid sequence analysis (GSA) and hierarchical clustering

As a secondary objective, we aim to identify distinct aggression dyad types within the complex and heterogeneous data in our reciprocal design. To achieve this, we employ the Grid Sequence Analysis (GSA) method introduced by Brinberg et al. (2016). The technique combines sequence analysis, commonly used in social science to capture time-ordered sequences (Abbott, 1983), with state-space grids (Lewis et al., 1999). The GSA enables the analysis of dyad-level time series and the identification of groups of similar dyad sequences. This approach allows us to investigate different types of sibling dynamics in terms of unique intra-dyad aggression patterns.

The general method used in the study was adjusted to our data based on the approach described by Brinberg and colleagues in 2016 (Brinberg et al., 2016). Within-dyad dynamics during the iTAP were represented as a joint trajectory in a two-dimensional state space grid. The 10 different aggression levels of the iTAP response scale were represented on a 10 × 10-dimension grid resulting in 100 cells. Each cell in the grid corresponded to a specific combination of aggression levels of both members of a dyad. Therefore, one dyad member was assigned to the horizontal-axis and the other member to the vertical-axis of the grid. We adjusted the GSA and employed an indistinguishable GSA version to capture dyadic dynamics across all interactive trials, while treating both siblings as interchangeable. We further applied the distinguishable GSA version, as described by Brinberg et al. (2016), for the fake trials. This original version allowed for an unambiguous role assignment and considered the pattern of the fake provocation and the responses of each participant individually, departing from the previous approach of analyzing data within a dyad structure. To simplify the explanation, we first describe the distinguishable version across the fake trials.

**FIGURE 2:**
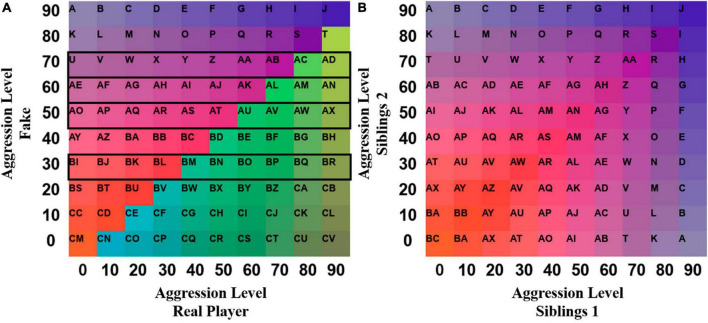
Depiction of the color-letter grids for the distinguishable and indistinguishable GSA approach. Each cell was labeled with successive letters or letter combinations from the English alphabet to handle sequence formation. The specific colors are chosen for visualization purposes only and have no innate relationship to the observation scale. **(A)** *Distinguishable pairs*: In this grid, the violet-orange color scheme represents the fake opponent having higher aggression scores than the actual player, while the blue-green scheme represents the opposite pattern. Due to the fixed nature of the five fake trials, only the scores on the highlighted rows were feasible. **(B)** *Indistinguishable pairs*: In this grid, combined trial-based observation scores between members are absolute, as the player ascribed to the x-axis and y-axis is interchangeable. Cell labels and colors are mirrored diagonally. Each base color corresponds to a different provocation level, and the shade indicates the relative aggression score of the other sibling. Shades containing a higher proportion of white are associated with a higher difference in aggression scores between siblings, while shades with a lower amount of white indicate more balanced aggression scores.

**FIGURE 3:**
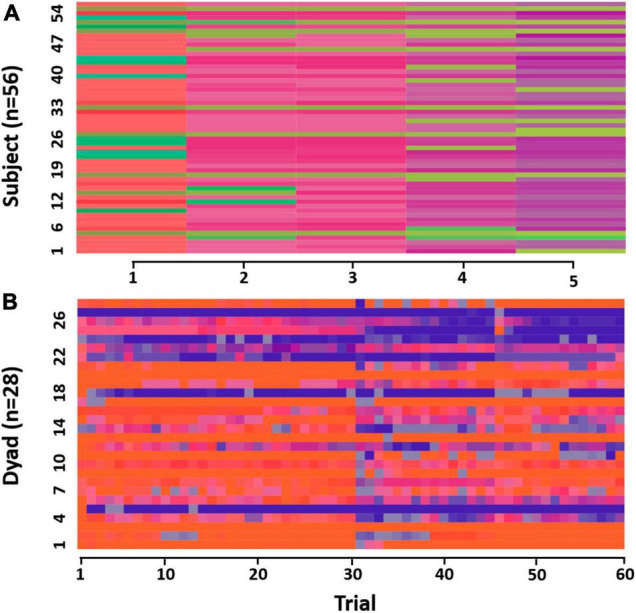
**(A)** Grid sequences depicting provocation aggression scores of all participants (*N* = 56) over trials (*N* = 5) as consecutively visited color-coded cells in the state space grid (see Figure 2A). **(B)** Grid sequences depicting interactive aggression scores of all dyads (*N* = 28) over time (*N* = 60) as consecutively visited color-coded cells in the state space grid (see Figure 2B). Fake trials occurring in the middle of the sequence were removed, resulting in a visible vertical line due to changes in interactive aggression scores.

For the distinguishable GSA, the real players were assigned to the x-axis and the fake player to the y-axis of the grid (Figure 2A). A composite score, referred to as provocation-aggression score, was created by combining the predefined provocation choices of the “fake player” with the actual choices made by all 56 participants. The provocation-aggression scores were captured as movement through the state space grid. Further analyses used the resulting sequences (Figure 3A).

For the indistinguishable version of the GSA, the assignment of members to either of the grid axes was not fixed across dyads, treating members as indistinguishable. To capture the interactive patterns, only the interactive trials were included in the analysis. Each interactive trial was assigned a letter-color combination, creating a trial-level dyad score for each dyad (Figure 2B). This composite score will further be referred to as interactive aggression scores. The state space was symmetrical at the diagonal and cells reflected at the diagonal were ascribed the same “absolute” interactive aggression score. An example trajectory of a dyad through the 10 × 10 grid is shown in Supplementary Figure 2 (all trajectories at^3^). Further analyses used the dyad-specific sequences (Figure 3B).

In the next stage of the GSA approach, distances between the dyad-level sequences were calculated. Therefore, a cost matrix was generated to define the costs of insertion, deletion and substitution between cells where insertion and deletion costs are set to 1 (substitution costs are automatically calculated based on these costs).

The costs in the matrix were determined based on the Manhattan Distance between cells, allowing lower costs for neighboring cells and stepwise increasing costs with each cell distance. Sequence analysis was then applied, and the costs were calculated to transform one sequence into all other sequences, considering the established cell-to-cell cost matrix. This was done using the Optimal Matching Algorithm. The result was a dissimilarity matrix of size n x n, which specifies the dissimilarity between each dyad and each other dyads (e.g., one row contains the dissimilarity of Dyad 1’s sequence with the sequences of Dyad 2 to Dyad 28).

Finally, the dissimilarity matrix was used as input for a hierarchical cluster analysis using the ward method. The length of the dendrogram branches and the number of dyads within each cluster were inspected to determine the number of clusters.

### 2.11 Cluster comparison: instruments and task ratings

The clusters resulting from the GSA (interactive trials) were compared across various aspects using Kruskal-Wallis tests. To validate the clusters and describe the aggression patterns within each cluster, we initially examined within-dyad mean and difference scores related to different iTAP scores (mean and difference scores across interactive and fake trials, frequency of “Zero Aggression Trials”, “Pre-Post Aggression Difference”). Second, we conducted comparisons of mean age, within-dyad age difference, and scores from chosen psychometric instruments, and task ratings across the identified clusters. We selected the psychometric constructs from existing literature and included measures of sibling relationship quality, along with broader constructs related to aggressive behavior. We thus tested trait physical aggressiveness, state anger, dominance motives, and sibling competition and mutuality. Task ratings were chosen to establish connections between siblings’ game perceptions and the identified behavioral clusters. These include subjective feelings of power after winning trials (WIN1), feelings of failure after losing trials (LOS1), motivation to win (competitiveness; MOT), perception of siblings’ dominant game behavior (TAP1), perception of siblings’ fairness (FAIR), and siblings’ intentional aggressive behavior (TAP7). The precise wording of the rating questions is provided in Supplementary Table 2 in the Supplementary materials under the corresponding label for reference.

A Chi-square test was conducted to assess the gender distribution across clusters. Bonferroni correction was applied to correct for multiple comparisons for seven iTAP scores (α* = 0.007). Dunn’s tests were conducted on significant results to perform pairwise comparisons between clusters. Further, due to the presence of collinearity among some of the predictors as well as the small cluster sizes, regression-based analyses could not be performed to compare the clusters across the iTAP and factor scores.

## 3 Results

### 3.1 Manipulation check and gender effects

**FIGURE 4:**
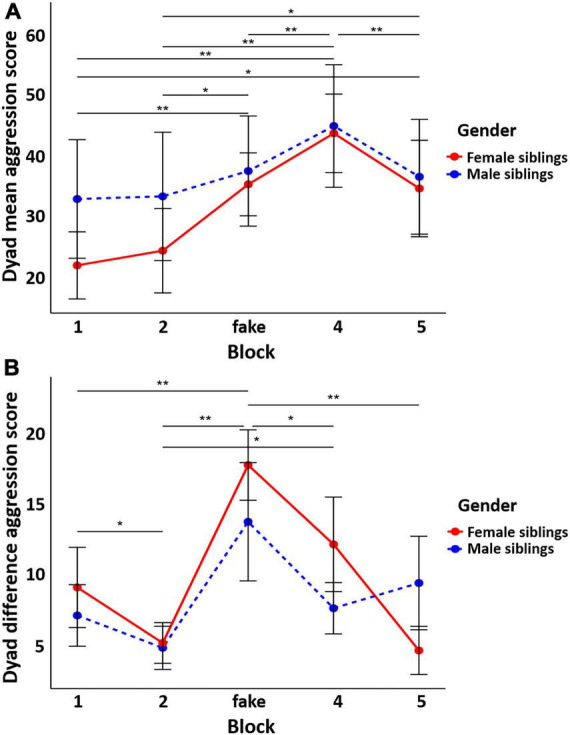
Dyad mean **(A)** and difference **(B)** aggression scores across blocks separately for male and female sibling pairs. No significant gender difference was found. Error bars represent the 95% confidence intervals around the mean values. * *p* < 0.05 and ** *p* < 0.01.

The results of the GEE analyses (see Figure 4) revealed a significant main effect of block for both dyad mean aggression scores [χ^2^(4) = 30.78, *p* < 0.001] and dyad difference aggression scores [χ^2^(4) = 35.99, *p* < 0.001]. The main effect for gender was not significant for dyad mean [χ^2^(1) = 0.29, *p* = 0.588] and dyad difference [χ^2^(1) = 0.06, *p* = 0.808] aggression scores. The interaction between gender and block on aggression scores was not significant [mean: χ^2^(4) = 4.75, *p* = 0.314, difference: χ^2^(4) = 6.93, *p* = 0.139]. *Post-hoc* tests (Supplementary Tables 6, 7) with Bonferroni correction showed significant differences in mean aggression scores between block 1 and block 3 (*p* < 0.001), 4 (*p* < 0.001) and 5 (*p* = 0.002). Block 2 showed significant differences with block 3 (*p* = 0.005) and 4 (*p* < 0.001). Block 3 demonstrated a significant difference with block 4 (*p* < 0.001) and block 4 with block 5 (*p* < 0.001). Regarding the direction of mean aggression scores, the preceding blocks consistently displayed lower mean aggression scores, except for the final block, which exhibited lower mean aggression scores compared to the preceding one. Regarding difference aggression scores, the fake block showed a significant difference with block 1, 2, and 5 (*p* < 0.001). Preceding blocks consistently showed higher difference in aggression scores until the fake block, with all following blocks displaying lower difference in aggression scores compared to the fake block.

The Kruskal-Wallis test on the aggression scores after wins, losses, and ties revealed a significant effect [*H*(2) = 6.58, *p* = 0.037]. Results of the pairwise comparisons using Dunn’s test did not survive correction for multiple testing (win vs. loss: *Z* = 2.36, *p* = 0.055; win vs. tie: *Z* = 2.07, *p* = 0.115; tie vs. loss: *Z* = −0.243, *p* > 0.999).

### 3.2 Aggression scores and correlates

Descriptive statistics, distribution details, and results of normality checks for mean aggression scores across all trials, interactive trials, and fake trials, along with the frequency of “Zero Aggression Trials,” are summarized in Supplementary Table 5. Information on dyads’ mean and difference aggression scores is also provided.

Additionally, Supplementary Table 3 presents descriptive statistics, distribution details, and normality check outcomes for all instruments and subscales employed in this study. Further, Cronbach’s Alpha coefficients are reported for each subscale, revealing poor internal consistency for all subscales of the BIS and NEO-FFI IV, and the longing subscale of the STQ.

In addition, Supplementary Table 3 reports the correlation analyses results between the three mean aggression scores and the instruments. Bonferroni correction was applied to account for multiple comparisons of all three mean aggression scores, adjusting the significance threshold (α = 0.05) separately for each subscale to α* = 0.017. Significant correlations surviving this adjusted threshold include a positive relationship between the state anger subscale of the STAXI and mean aggression scores across all trials [ρ(56) = 0.36, *p* = 0.006] and all interactive trials [ρ(56) = 0.37, *p* = 0.005]. Additionally, the extraversion subscale of the NEO-FFI IV positively correlates with the mean aggression scores during fake trials [ρ(56) = 0.35, *p* = 0.008]. Given the poor internal consistency of the extraversion subscale, this result should be interpreted with caution, and will not be further referenced.

Dyad-level correlations between mean and difference scores of task aggression and subscale scores are reported in Supplementary Table 4 together with descriptives of all dyad mean and difference subscale scores. Bonferroni correction was applied to account for the 12 tests conducted per subscale (mean and difference of subscale scores for the three task scores). The adjusted alpha was set to α* = 0.004, and only one dyad difference score survived – a negative correlation between the within-dyad difference on the longing subscale of the STQ and the difference of all three aggression scores [all: ρ(56) = −0.71, *p* < 0.001, interactive: ρ(56) = −0.62, *p* < 0.001, fake: ρ(56) = −0.67, *p* < 0.001]. We refrain from further interpretation of this result due to poor internal consistency observed in the subscale longing of the STQ.

## 3.3 GSA

### 3.3.1 Hierarchical clustering analysis – interactive trials

**FIGURE 5:**
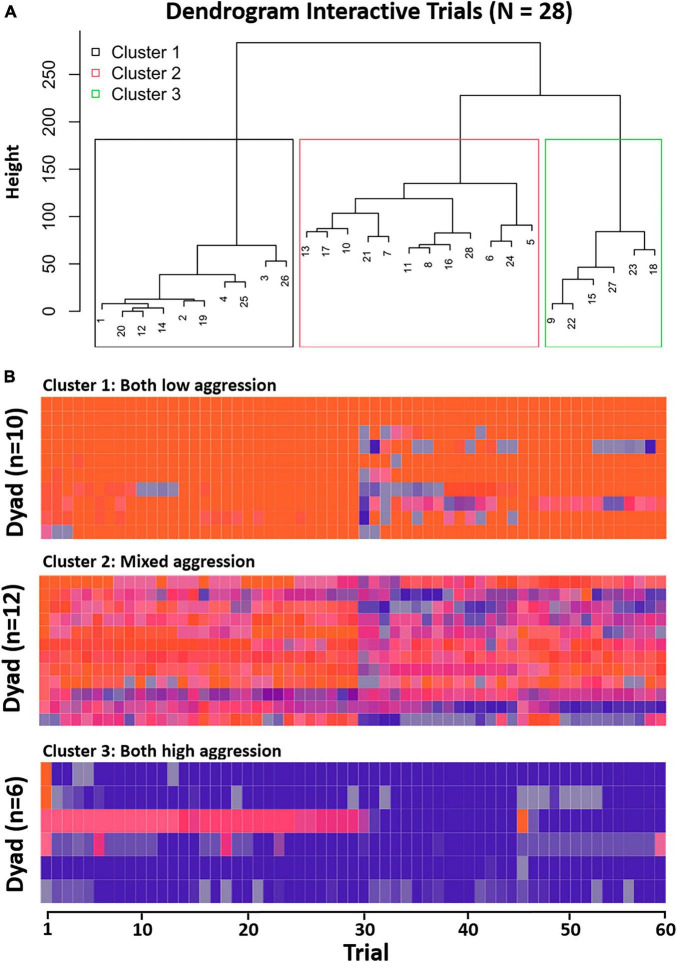
**(A)** Dendrogram interactive trials (distinguishable). **(B)** Sequences per cluster depicting interactive aggression scores over trials (from left to right). Cluster labels are for description only.

Hierarchical clustering of the interactive aggression score sequences revealed a three-cluster solution (Figure 5).

**FIGURE 6:**
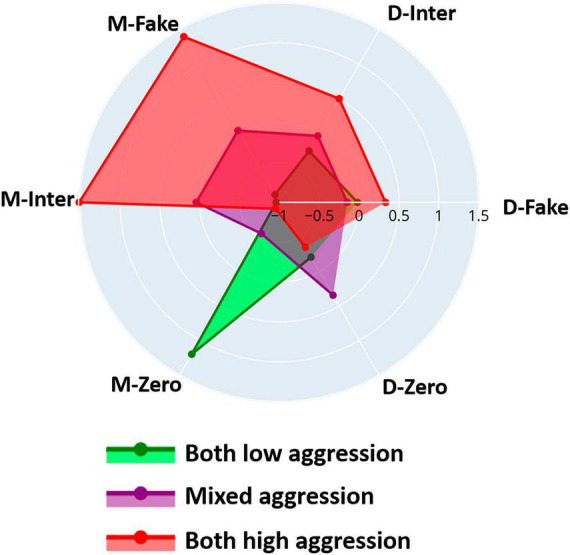
Radar plot depicting differences in various aggression scores across the three clusters: “Both low aggression,” “Mixed aggression,” and “Both high aggression” derived from clustering differences in interactive aggression score trajectories: Normalized dyad mean aggression scores for interactive (M-Inter) and fake (M-Fake) trials, frequency of zero-aggression score selection (M-Zero), and within-sibling variability of these variables (D-Inter, D-Fake, D-Zero, respectively).

The resulting clusters showed distinct patterns in various aggression scores, including dyad mean and difference aggression scores and the mean frequency of selecting the zero-aggression score (visualized in a radar plot in Figure 6). The clusters also showed a significant difference in mean age, while no significant differences were observed in the age difference between siblings or gender distribution within the clusters. A summary table of the three clusters in terms of these iTAP score characteristics, as well as demographics is given in Supplementary Table 8. Further results of Kruskal-Wallis tests and *post-hoc* tests of the iTAP scores are given in Supplementary Table 9.

### 3.3.2 Cluster differences across instruments and task ratings

**FIGURE 7:**
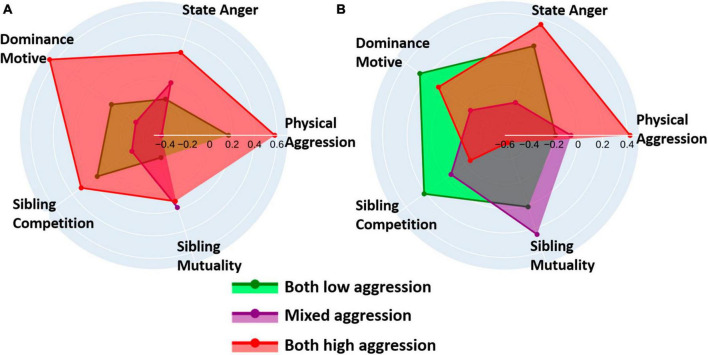
Radar plots depict **(A)** normalized mean scores of the five instrument subscales [physical aggression (BPAQ), state anger (STAXI), dominance motive (DoPL), competition (STQ), and mutuality (STQ)] within each cluster, and **(B)** the difference scores of normalized mean scores between siblings within each pair across the clusters.

**FIGURE 8:**
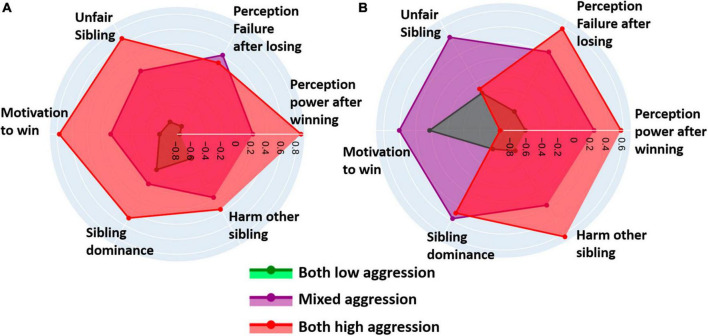
Radar plots display **(A)** normalized mean scores of the six task ratings [Perception of power after winning (WIN1), Perception of failure after losing (LOS1), Unfair sibling (FAIR), Motivation to win (MOT), Sibling dominance (TAP1), and Harm other sibling (TAP7)] within each cluster, and **(B)** the difference scores of normalized mean scores between siblings within each pair across the clusters.

Dyad mean and difference scores of z-normalized cluster averages across selected instrument subscales and task ratings are visualized in four radar plots: dyad mean and difference scores for the scores on the instrument subscales (see Figure 7) and the task ratings (see Figure 8) separately. Kruskal-Wallis tests comparing the clusters across the instrument subscales revealed no significant difference for any mean and difference score. Concerning task rating, significant differences for mean and differences of emotional state task ratings were found, specifically in feelings of power after winning [mean: *H*(2) = 13.34, *p* = 0.001; difference: *H*(2) = 9.89, *p* = 0.007], and feelings of failure after losing [mean: *H*(2) = 8.58, *p* = 0.014; difference: *H*(2) = 7.82, *p* = 0.020]. Additionally, significant differences were observed in mean and difference scores in overall perception of sibling’s fairness across clusters [mean: *H*(2) = 7.80, *p* = 0.020; difference: *H*(2) = 6.60, *p* = 0.037]. The cluster effect was significant for the difference scores of sibling’s winning motivation [*H*(2) = 7.38, *p* = 0.025], of perception of siblings’ dominant game behavior [*H*(2) = 7.00, *p* = 0.030], and of siblings’ intentional aggressive behavior [*H*(2) = 7.14, *p* = 0.028]. Clusters did not differ regarding mean sibling scores. Complete test statistics for the cluster comparison and *post-hoc* tests are provided in Supplementary Table 10.

### 3.3.3 Hierarchical clustering analysis – fake trials

The hierarchical clustering of the provocation-aggression scores (see Figure 3A) resulted in the identification of three distinct clusters (see Supplementary Figure 3). These fake trial clusters exhibited variations in the mean and standard deviation of the real players’ aggression scores (see Supplementary Figure 4). Cluster 1 was characterized by higher fake aggression scores than actual player aggression scores, with siblings exhibiting a mean aggression score of 14.2 (*SD* = 16.25). In Cluster 2, siblings displayed a mean aggression score of 87.50 (*SD* = 5.89) and this was the only cluster characterized by higher participants’ real aggression scores than fake aggression scores. Cluster 3 showed higher aggression scores for the fake opponent and mean aggression scores that fell in the mid-range for the siblings, with a mean aggression score of 42.43 (*SD* = 8.98). Siblings within the same dyad, mostly fell into the same fake-trial clusters, with only 5 out of 28 sibling pairs falling into different individual clusters.

## 4 Discussion

The present study aimed to evaluate the effectiveness of the newly developed interactive Taylor Aggression Paradigm (iTAP) to capture bidirectional provocation-induced aggression in sibling relationships. Our primary focus is on examining the behavioral dynamics of siblings’ reciprocal reaction to provocation and identification of naturally occurring sibling types in terms of aggression patterns. We further discuss underlying personal and situational factors that influence these dynamics.

For the iTAP to serve as an effective tool to capture reactive aggression, it is crucial that it robustly responds to situational factors that escalate aggression. As predicted, in comparison to the real interactions, we observed a rise in aggression during the manipulated trials. These trials included a steady elevation in the provocation level which was for most sibling pairs higher than the starting aggression level. Siblings’ responses to perceived provocation thus largely align with findings from TAP studies using fake provocation that showed an increase in aggression proportional to provocation levels (Weidler et al., 2019; Konzok et al., 2020). In the present study, the aggression further escalated during the real interaction between siblings after the manipulated trials suggesting the initiation of an aggression helix. Interestingly, comparably lower aggression levels during the final block indicated a reciprocal decline in aggression probably reflecting a de-escalation. The reduction in variability between siblings’ amount of monetary punishment at this point further suggests a convergence in their decision. These dynamics align with “tit-for-tat” decision-making commonly observed in the study of laboratory-induced reactive aggression. This is high provocations leading to retaliation involving high aggression, and low or no provocations leading to low or no aggressive responses (Chester, 2017; Dinić and Smederevac, 2019). Interestingly, on average dyads in the present study autonomously de-escalated their aggression levels, during genuine interaction, suggesting inherent regulatory mechanisms within the behavioral dynamics of the sibling pairs. These behavioral regulation strategies can be related to the concept of emotional coregulation within close relationships (Butler and Randall, 2013). In the sibling relationship during development, successful co-regulation between siblings has been linked to siblings’ positive psychosocial outcome (Paley and Hajal, 2022). In contrast, co-regulation can become dysregulated in close relationships of clinical populations; for example, patients with borderline personality disorder have been found to use ineffective co-regulation strategies (Miano et al., 2021). Therefore, we would expect different patterns of reciprocal behavior after the induced provocation in clinical samples.

Additionally, sibling aggression levels were further influenced by the game outcome, which is consistent with previous findings in monetary TAP studies (Weidler et al., 2019; Boccadoro et al., 2021). However, it remains unclear whether this effect is specific to the use of money as punishment modality. Up to the author’s knowledge, this is also the first time the rock paper scissor game was employed in the study of aggression and improved the credibility of the cover story. Specific effects on reactive aggression remain to be discovered.

The results demonstrate that the iTAP can capture both provocation-induced variations in reactive aggression as well as the natural development of aggression occurring in genuine interactions as evident in the adjustment process after induced provocation. We also emphasize that the game outcome as potential component of frustration plays an important role in the real interaction−although in the current paradigm there was no performance component involved as in some previous TAP versions.

Previous studies have consistently reported gender differences in the overall aggression level during the TAP (Weidler et al., 2019; Konzok et al., 2020). Although the gender differences observed in our study did not reach statistical significance, we noted similar trends that align with our initial hypothesis. Brother pairs initially chose higher levels of aggression compared to sister pairs. However, brother and sister pairs showed a convergence in their aggression score during and after the fake trials, indicating a shift toward more similar levels of aggression after provocation. It is necessary to replicate the study with a larger sample to determine if those gender-related trends in aggression on the iTAP are generalizable. Furthermore, it is necessary to investigate whether the observed trend in gender differences persists in non-sibling pairs and opposite-sex pairs or is specific to dynamics within same-sex sibling pairs. Other evidence supporting the influence of sibling gender on aggression dynamics comes from a study on aggressive behavior of twins which used the TAP procedure (Dinić et al., 2020). Sisters from opposite-sex twin pairs exhibited more aggression toward their male twin counterparts compared to women in same-sex dyads from the control group of strangers.

Supporting the ecological validity of the iTAP, the mean aggression scores were positively correlated with participants’ levels of reported state anger. This is in line with the notion, that anger facilitates aggression in a provocation context (Winstok, 2007). Nevertheless, the interpretation of this relationship should be considered within the context of the small pilot sample size and remains subject to further validation. In contrast, we did not find the expected positive associations between aggression scores on the iTAP and more stable personality traits that are commonly associated with aggressive behavior, including trait aggressiveness (Krämer et al., 2008), or impulsivity (Moore et al., 2022). Reasons for the absence of significant associations may be that our sample included participants with low trait aggressiveness, and the small sample size limiting variance in the subscales used. Nevertheless, the task may have elicited a temporary state of frustration, leading to increased levels of situational anger and therefore aggression across participants independent of their traits. In line with this, a previous study found increased levels of state anger after a provocation task (Repple et al., 2017).

We identified three subgroups comparing the dyadic aggression courses in the iTAP. Consistent with our initial hypothesis, these subgroups were characterized as employing either a fairness strategy or a more competitive strategy based on the aggression patterns. In both subgroups, aggression scores tended to be predominantly either mutually low or mutually high throughout the trials. A third cluster exhibited a more mixed aggression pattern, with varying aggression scores in the task and the strongest reaction to the manipulated aggression trials. Future research should explore the replicability of these subgroups to determine if they reflect a general trend or if they are specific to same-sex young adult siblings. Sibling relationships are complex, as siblings possess the potential for both competition, e.g., competing for parental resources such as attention, care, and money (Salmon and Hehman, 2015) and cooperation as suggested by Hamilton’s kin selection theory (Hamilton, 1964; Neyer and Lang, 2003). Consequently, finding that high aggression and low aggression subgroups align with the dual nature of sibling relationships highlights the option for cooperation and competition behavioral patterns in the iTAP. It is crucial to acknowledge that participants were explicitly instructed about the competitive nature of the game, potentially influencing the strategic decisions made by the siblings (Meyer et al., 2012).

Each subtype was characterized by distinct profiles related to personal factors and situational task ratings. It is essential to note that associations with personal factors are preliminary and serve as a descriptive overview in this study. A more in-depth interpretation is reserved for future investigations with larger samples sizes.

The high aggression subgroup exhibited, as anticipated, the highest total levels of physical aggression, state anger, dominance, and sibling competition among its members. Surprisingly, they also scored the highest on sibling mutuality, measuring the degree of closeness, understanding, and similarity in crucial life aspects within sibling relationships (Szymańska, 2016). Siblings within this cluster differed in physical aggression and state anger, and, to a lesser degree, in dominance, but not much in measures of sibling relationship quality. Task experience ratings aligned with siblings’ behavior on the task. They felt the most powerful after winning trials, especially when compared to the low aggression cluster, and reported the highest motivation to win the game. Siblings in this cluster were most aligned in perceiving their sibling as unfair, while they showed the greatest discrepancy in how dominant they perceived their sibling during the task compared to the other clusters.

The low aggression subgroup showed moderate scores on the personal measures. They had lower mean sibling competition scores, but higher sibling difference scores in dominance and sibling mutuality, compared to the other clusters. Siblings in low aggression clusters also reported the least feelings of failure after losing and power after winning trials, while exhibiting the least difference between these emotional states. Evaluation of the other sibling was fairer than in the other clusters and they reported the least motivation to win. Siblings agreed on how dominant they perceived the other sibling and agreed in their motivation to harm their sibling.

The mixed aggression subgroup exhibited high levels of sibling competition, while showing the largest within-pair difference on this relationship dimension. This could imply that in the mixed aggression subgroup, one sibling evaluated the sibling relationship as more competitive than their counterpart. This dynamic may have influenced the mixed aggression pattern, with the more competitive oriented sibling selecting higher amounts of money and the less competitive sibling attempting de-escalation by choosing lower amounts. In line with this, the subgroup behaved with higher aggression after the external provocation block. This subgroup also reported greater feelings of power after winning trials, failure after losing trials, and evaluation of the other sibling as unfair most of the time compared to the low aggression subgroup. However, the dissimilarity of siblings in the mixed cluster becomes evident when comparing the ratings between siblings as they showed high differences on all ratings. Especially the motivation to win, the perception of unfairness, and how dominate they viewed the others’ game behavior were deviating.

Interestingly, in the same sample using other interactive tasks, we observed that sibling pairs exhibited high dominance traits displayed more competitive behavior, and a relatively apathetic sibling relationship. Siblings with low dominance traits showed a more affectionate and reciprocal relationship and cooperative turn-taking strategies during an interactive version of the Chicken Game task (Hernandez-Pena et al., 2023). In the iTAP, sibling dominance and motivation to win were associated with a mutual aggression strategy. Preliminary results of personality factors hint to a higher level of dominance traits in sibling pairs utilizing this strategy compared to strategies found in the other clusters. A previous study suggested that sibling aggression is associated with intimacy, negativity, and time spend together (Updegraff et al., 2005). Nevertheless, we did not identify a significant association between sibling relationship quality and a more dominant task behavior of the siblings.

Regarding demographic differences, the subgroups did not differ in gender distribution or age difference between siblings within a pair. However, the mixed aggression subgroup consisted of younger individuals than the other subgroups.

Theories on aggression such as the General Aggression Model (Anderson and Bushman, 2002) postulate an influence of situational and personal variables on the likelihood of aggression to emerge. Although, we could not robustly associate personal variables with levels of aggression in the iTAP, our findings highlight the influence of situational triggers on aggression escalation. Sibling aggression levels were influenced by provocation levels and game outcomes, with a potential mediation through anger.

Sibling pairs, on average, modulated their behavior in response to external provocation and their partners behavior. This finding is consistent with previous research by Anderson et al. (2008) who found that high initial aggression at the start of a task results in sustained high aggression throughout the task. Extending this idea, our findings suggest that not only high aggression, but also low aggression levels can be reciprocated and form stable patterns over time. This highlights the often reported finding of a “tit-for-tat” strategy employed in the TAP (Krämer et al., 2011; Wagels et al., 2018; Weidler et al., 2019). Comparing the overall sibling behavior across trials and behavioral profiles of subgroups, we further observed that pairs exhibit different but consistent patterns throughout the task modulating these general “tit-for-tat strategies” into other forms of reciprocal behavior. It appears that some players settle into a particular level of punishment with their opponent. When considering TAP versions with an ostensible opponent, this inflexibility of the player can critically influence the findings. Lastly, it is also important to note the correspondence in aggression scores between the interactive and fake clusters, indicating that siblings did not diverge on the overall pattern at the dyad level during the fake trials but differed primarily in absolute mean value.

## 5 Limitations and strengths

Some limitations of our study should be acknowledged. First, our sample size was small as it was part of a pilot study, which may limit the generalizability of the findings, especially when examining differences across clusters with small cluster sizes. In line with this, certain subscales exhibited poor internal consistency, posing challenges to interfering relationships between task behavior and those constructs measured. Second, we did not include variables that access current within-family effects such as parental involvement and attachment or family support and cohesion which could provide further insights into sibling dynamics. Our sample consisted of siblings who voluntarily participated, which implies that they were likely in contact and on good enough terms, potentially affecting the generalizability of the results to the wider population of siblings. The dynamics observed in our study may not be fully generalizable to siblings who have more strained or distant relationships. Likewise, since our study focused on gender effects within same-sex sibling pairs, the findings might not be generalizable to different dyads and gender constellations and neither to other populations or types of dyadic relationships. To enhance the external validity of our findings, it would be valuable to test the paradigm in different populations, such as friendships or romantic relationships. A control group, e.g., strangers, would be advisable. Additionally, while our study concentrated on a stable and developmentally homogeneous age group of young adult siblings, exploring the dynamics among child siblings would be intriguing. Our current study served as a pilot for a hyperscanning fMRI study, and children were not included as a sample group due to the study’s focus and design. Third, there could be potential carry-over effects of previous tasks, particularly given the decision to cooperate or compete in the interactive Chicken Game. Finally, it is important to acknowledge the potential presence of common method bias, as most of the instruments were administered in a test battery at the end of the study, which might have influenced the measured relationships.

We emphasis the validity of the new paradigm regarding the ecological aspect of the monetary TAP that connects to sibling competition over resources. Although sibling relationships become more harmonious with age, siblings still compete in areas including socioeconomic status and general success in life (Sumijati and Widhi, 2018). Moreover, we propose that the iTAP can serve as a valuable tool for examining aggressive social dynamics of clinical populations in close relationships, including family members. Reactive aggression is notably heightened across various patient groups characterized by threat hypersensitivity, frustration, and hostility bias, such as borderline personality disorder or antisocial personality disorder. Utilizing the iTAP can contribute to a better understanding of symptomatology and potentially inform therapeutic interventions.

## 6 Conclusion

The present study demonstrates the effectiveness of the novel interactive Taylor Aggression Paradigm (iTAP) to examine sibling interaction dynamics within a provocation context. The results showed that in healthy (low aggression) siblings, aggression levels can increase due to short provocation induction, but decrease via subsequent co-regulation between siblings. Aggressive behavior in the iTAP is positively associated with state anger across siblings. Different sibling dyad types can be characterized by unique provocation-aggression patterns and self-described dominance as well as perception of superiority and fairness in the iTAP. The study supports the validity of the iTAP as a valuable tool for studying aggression within sibling relationships in a more naturalistic setting, underpinned by the emergence of different types of reciprocal aggression patterns.

## Data availability statement

The datasets presented in this study can be found in online repositories. The names of the repository/repositories and accession number(s) can be found below: https://osf.io/f9gvz/?view_only=bcbe206a4b6a437396b5ed417313ffaf.

## Ethics statement

The studies involving humans were approved by the Ethik-Kommission an der Medizinischen Fakultät der RWTH Aachen and Center for Clinical Trials (“Studying the intra- and inter-brain underpinnings of aggression, dominance, and conflict”; CTC-A Nummer 20-485; Internes Aktenzeichen EK 407/20). The studies were conducted in accordance with the local legislation and institutional requirements. The participants provided their written informed consent to participate in this study.

## Author contributions

JK: Data curation, Formal analysis, Investigation, Methodology, Project administration, Visualization, Writing− original draft, Writing−review and editing. LH-P: Conceptualization, Data curation, Investigation, Project administration, Software, Writing−review and editing. CK: Data curation, Investigation, Writing−review and editing. EB: Writing−review and editing. UH: Funding acquisition, Writing−review and editing. RS: Software, Writing−review and editing. LW: Conceptualization, Funding acquisition, Supervision, Writing−review and editing.

## Footnotes

1
https://osf.io/qdevj

2
www.soscisurvey.de

3
https://osf.io/f9gvz/?view_only=bcbe206a4b6a437396b5ed417313ffaf
