# Supplementary material for: A quasi-experimental study in sibling dyads: differential provocation-aggression patterns in the interactive taylor aggression paradigm
Source: Front Psychol. 2024 Feb 8;15:1288743. doi: 10.3389/fpsyg.2024.1288743 (PMC10881662; doi:10.3389/fpsyg.2024.1288743)
Supplement: Supplementary file 1 [file Data_Sheet_1.docx]

Supplementary Material

# Supplementary Methods

## **Server Communication**

Task synchronization was managed by a virtual server hosted at the Brain Imaging Facility of the Interdisciplinary Center for Clinical Research (IZKF Aachen) and implemented with the socket module in Python 3.6.6. Client-server communication was handled via Transmission Control Protocol (TCP)/Internet Protocol (IP). The clients, i.e., task-presenting computer of both participants, send task-specific information to the server. Upon receiving both signals, the server sends a trigger back to the clients, initiating the tasks simultaneously in PsychoPy and managing ongoing synchronization and communication during the task.

# Supplementary Figures

##
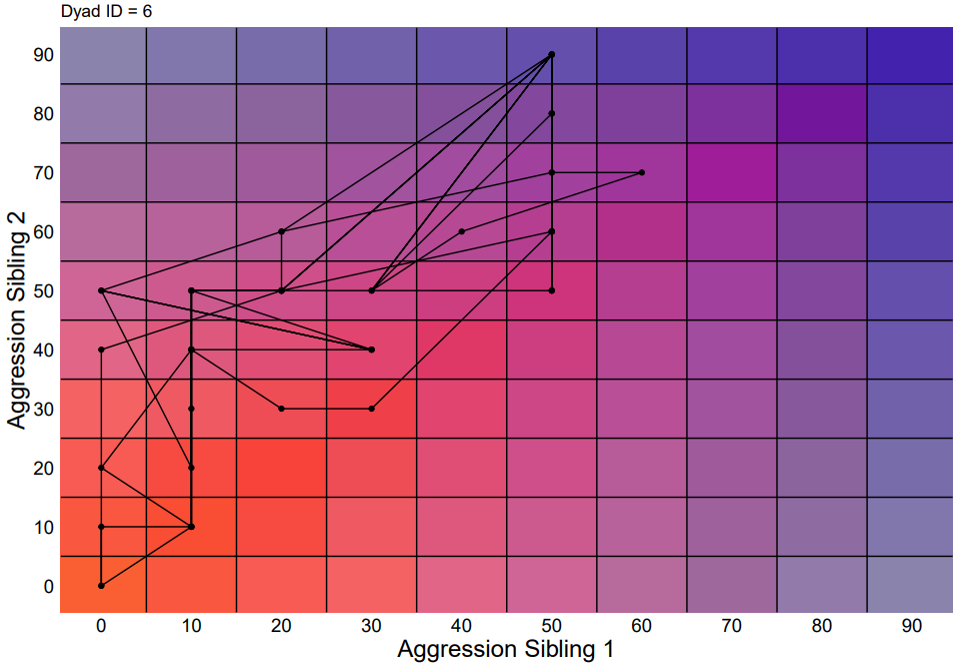


**Figure S2**. Example trajectory of a dyad through the state space grid of the indistinguishable GSA.

## Fake trials


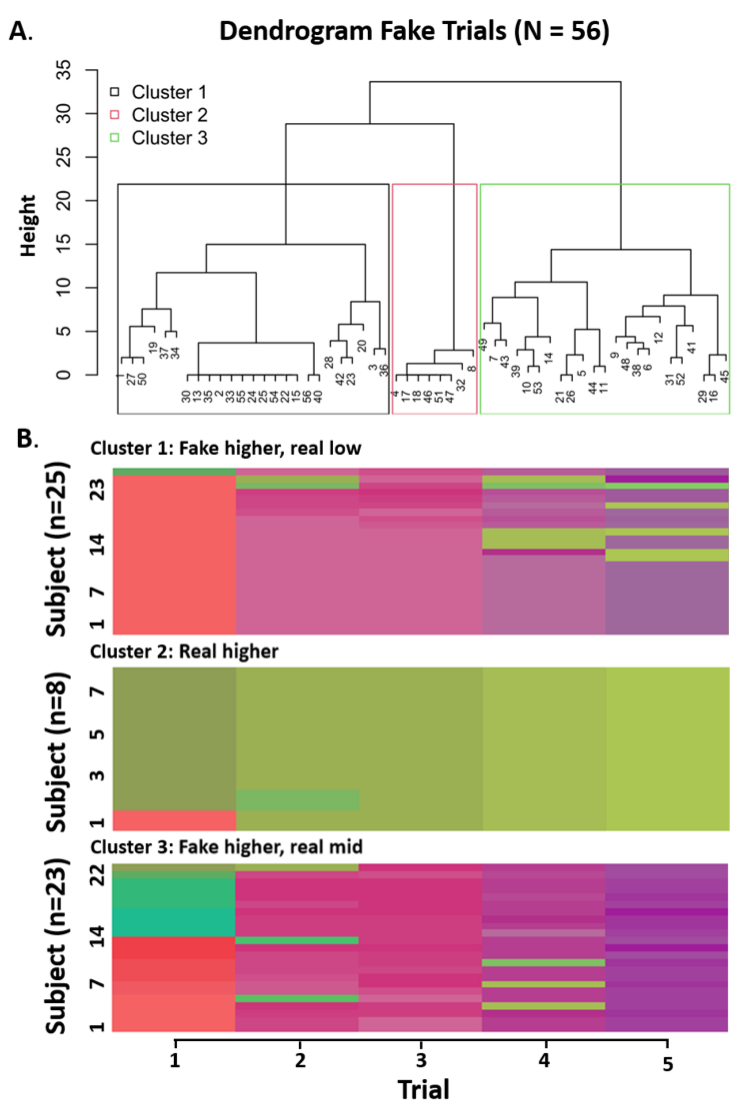


**Figure S3**. Dendrogram of the hierarchical clustering of the distance matrix of provocation-aggression scores **(A)**. Sequences divided by cluster. Cluster labels are for description only. The violet-orange color scheme represents the fake opponent having higher aggression scores than the actual player, while the blue-green scheme represents the opposite pattern **(B)**.


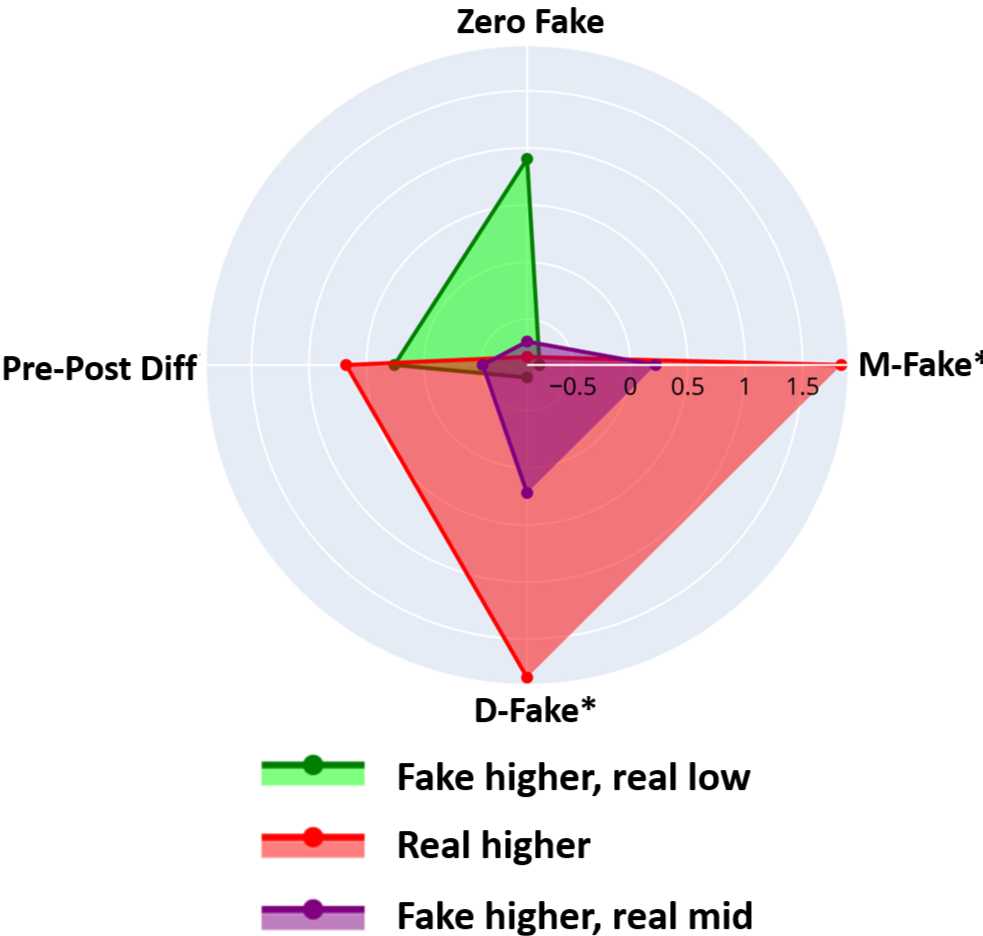


**Figure S4.** Radar plot illustrating variations in z-standardized mean aggression scores across fake trials, difference from manipulated provocation score mean, frequency of zero-aggression score selection during fake trials, and difference between mean aggression score before and after the fake block across the three clusters: "Fake higher, real low", "Real higher", and "Fake higher, real mid" derived from clustering differences in provocation-aggression score trajectories. *Note*. *across all *N* = 56 siblings individually.

# Supplementary Tables

## **Ratings and Variable Definitions**

**Table S1**. Rating scales created for the iTAP game assessing feelings regarding won and lost trials, as well as behavioral motivations after each block and general opinions about the game and the players’ behavior after the task. Abbreviations were used throughout the paper when referring to specific ratings.

| **Abbr.** | **Rating** |
| --- | --- |
|  | Question at the end of the task |
|  | 1. Statements - how much does this describe the game (1=Little, 5=A lot): |
| TAP1 | "My sibling tries to dominate the game." |
| TAP2 | "My sibling tries to harm me." |
| TAP3 | "My sibling does not dare to be mean." |
| TAP4 | "I do not dare to be mean." |
| TAP5 | "If I am treated fair I am fair, too." |
| TAP6 | "We try to achieve a similar gain for both." |
| TAP7 | "I try to harm my sibling." |
| TAP8 | "I dominate the game." |
| TAP9 | "I adjust to my sibling’s behavior." |
|  | **Questions between blocks** |
|  | 1. **Motivation** - In the last block I had the following thoughts (0-100): |
| MOT | "It didn’t matter who won." (0) - "I wanted to win." (100) |
|  | 2. **Satisfaction** - What did you like most about winning? (0-100) |
| SAT | "That I won money." (0) - "That my sibling lost money." (100) |
|  | 3. **Money** - If you withdrew more money, the reason was most likely (0-100): |
| MON | "I wanted to increase my chances of winning." (0) -"I wanted to harm my sibling." (100) |
|  | 4. **Fairness** - How fair was your sibling? (1-5) |
| FAIR | 1. all round (100%), 2. most (75%), 3. half (50%), 4. a few (25%), 5. never (0%) |
|  | 3. **Win questions** - Please rate your feeling after WINNING a round (1=Not at all, 5=Extremely): |
| WIN1 | "I felt powerful." |
| WIN2 | "I felt superior." |
| WIN3 | "I was afraid of revenge." |
| WIN4 | "I felt happy." |
| WIN5 | "I felt guilty." |
| WIN6 | "I felt dominant." |
| WIN7 | "I felt like I was fair." |
|  | 4. **Lose questions** - Please rate your feeling after LOSING a round (1=Not at all, 5=Extremely): |
| LOS1 | "I felt like I was failing." |
| LOS2 | "I felt inferior." |
| LOS3 | "I felt angry." |
| LOS4 | "I felt vindictive." |
| LOS5 | "I was happy for my sibling." |
| LOS6 | "I was sad." |
| LOS7 | "I had the feeling it served me right." |

**Table S2**. Variable description of all iTAP scores used thought the analyses.

| Abbr.^a^ | Analysis | Level | In-text name | Description |
| --- | --- | --- | --- | --- |
| TAP all  TAP interactive  TAP fake | Corre-  lation,  WSRT | Ind. | Mean aggression score  - All trials  - Interactive trials  - Fake trials | Individual-wise mean monetary selections (aggression scores) were calculated across all trials, all interactive trials and all fake trials separately. |
|  | KW (outcome) | Ind. | Aggression scores after wins, losses, and ties | Individual aggression scores were grouped and averaged based on their preceding game outcome (win, loss or tie). |
| (TAP) Zero |  | Ind. | “Zero Aggression Trials” | Frequency of zero-aggression score selection during all trials. |
| M-Fake*,  D-Fake* | KW  (cluster fake) | Ind. | Mean aggression scores across fake trials  Difference mean aggression score and manipulated provocation score mean | Individual mean aggression scores across all fake trials.  Difference between the individual mean aggression scores across all fake trials and the manipulated provocation score mean. |
| Zero Fake* | KW  (cluster fake) | Ind. | “Zero Aggression Fake Trials” | Frequency of zero-aggression score selection during fake trials. |
|  | GEE | Dyad | Dyad mean aggression score  Dyad difference aggression score | Difference and mean aggression scores between the two members of each sibling pair were computed separately for each block. Averages of these scores across all dyads and blocks were calculated separately for brother and sister pairs. |
| M-Inter,  M-Fake | KW  (cluster) | Dyad | Dyad mean aggression score | Per dyad individual mean aggression scores are averaged over all interactive trials and all fake trials separately. |
| D-Inter,  D-Fake | KW  (cluster) | Dyad | Dyad difference aggression score | Per dyad a difference score of members’ individual mean aggression score is calculated separately for mean aggression scores over all interactive trials and all fake trials. |
| Pre-Post Diff | KW  (cluster) | Ind./Dyad | “Pre-Post Aggression Difference” | Difference score of mean aggression after fake block and mean aggression before fake block. |
| M-zero,  D-zero | KW  (cluster) | Dyad | Mean and difference frequency zero-aggression score | Mean frequency of zero-aggression score selection per dyad and difference between dyad members in the frequency of zero-aggression score selection. |
|  | GSA | Dyad | Interactive aggression score | Trial-wise composite score of both dyad members aggression scores. |
|  | GSA | Ind. | Provocation-aggression score | Composite score for all fake trials of real players’ aggression score and manipulated provocation score. |

*Note*. ^a^Abbreviation used in Figures or Tables; *across all *N* = 56 siblings individually; WSRT = Wilcoxon signed-rank test; KW = Kruskal-Wallis; GEE = Generalized Estimating Equations; GSA = Grid Sequence Analysis.

## **Descriptives, Distributions, and Correlations**

- **Table S3**. Table including descriptives, distribution information and internal consistency of all instruments and subscales as well as correlation with individual mean iTAP scores.

| Variable/ Scale | Descriptives | | Correlation  Mean TAP across trials: | | | Shapiro-Wilks | Distribution | | Reliabil-ity |
| --- | --- | --- | --- | --- | --- | --- | --- | --- | --- |
|  | *M* | *SD* | All (*n*=65) | Inter. (*n*=60) | Fake (*n*=5) | W | Sk | Ku | CA |
| **Age** | 23.39 | 3.42 | -0.14 | -0.14 | -0.11 | 0.95 | 0.72 | 0.32 | - |
| **BPAQ** |  |  |  |  |  |  |  |  |  |
| Physical Aggression | 15.35 | 5.67 | 0.16 | 0.15 | 0.17 | 0.89 | 1.01 | 0.27 | .78 |
| Verbal Aggression^a^ | 13.57 | 2.92 | 0.08 | 0.08 | 0.07 | 0.98 | 0.32 | 0.29 | .56 |
| Anger | 14.80 | 5.13 | 0.04 | 0.04 | 0.02 | 0.91 | 0.94 | 0.38 | .72 |
| Mistrust^a^ | 18.14 | 4.45 | 0.09 | 0.09 | 0.05 | 0.99 | 0.25 | -0.14 | .32 |
| **RSPQ** |  |  |  |  |  |  |  |  |  |
| DL^a^ | 18.52 | 4.06 | 0.05 | 0.04 | 0.09 | 0.98 | -0.74 | 0.72 | .86 |
| CB | 28.63 | 3.44 | -0.13 | -0.13 | -0.04 | 0.96 | -0.13 | -0.31 | .75 |
| RSA | 11.27 | 3.88 | -0.13 | -0.13 | 0.11 | 0.94 | 1.02 | 1.65 | .74 |
| **DoPL** |  |  |  |  |  |  |  |  |  |
| Dominance^a^ | 23.73 | 6.07 | 0.09 | 0.08 | 0.05 | 0.97 | 0.04 | -0.75 | .80 |
| Prestige^a^ | 42.11 | 7.28 | 0.21 | 0.20 | 0.22 | 0.98 | -0.36 | 0.34 | .84 |
| Leadership^a^ | 38.00 | 8.23 | -0.01 | -0.02 | 0.02 | 0.98 | -0.14 | -0.34 | .87 |
| **HCA** | 64.26 | 10.40 | 0.09 | 0.10 | 0.03 | 0.99 | 0.04 | -0.26 | .61 |
| **NEO-FFI** |  |  |  |  |  |  |  |  |  |
| O^a^ | 37.40 | 5.03 | -0.22 | -0.22 | -0.19 | 0.97 | -0.45 | -0.29 | .42 |
| C^a^ | 38.84 | 3.40 | 0.15 | 0.14 | 0.13 | 0.98 | -0.22 | -0.38 | .43 |
| E^a^ | 33.07 | 4.88 | 0.32* | 0.32* | 0.35** | 0.97 | 0.00 | -0.84 | .56 |
| A^a^ | 42.45 | 4.22 | 0.09 | 0.09 | 0.11 | 0.97 | -0.10 | -0.52 | .40 |
| N^a^ | 33.55 | 4.88 | -0.17 | -0.17 | -0.25 | 0.97 | 0.44 | -0.44 | .44 |
| **PDCA** | 52.71 | 9.65 | 0.16 | 0.14 | 0.22 | 0.96 | -0.54 | -0.39 | .85 |
| **SoPS** | 4.33 | 0.67 | -0.09 | -0.09 | -0.01 | 0.91 | -1.13 | 1.33 | .82 |
| **STAXI** |  |  |  |  |  |  |  |  |  |
| Trait | 16.75 | 4.08 | 0.13 | 0.13 | 0.06 | 0.93 | 0.87 | 0.32 | .74 |
| State | 12.00 | 5.02 | **0.36**** | **0.37**** | 0.25 | 0.44 | 3.53 | 12.55 | .94 |
| In | 16.05 | 5.01 | 0.10 | 0.11 | 0.04 | 0.95 | 0.68 | 0.30 | .83 |
| Out | 12.13 | 4.04 | 0.14 | 0.17 | 0.09 | 0.86 | 1.42 | 1.87 | .80 |
| Control^a^ | 24.00 | 3.74 | -0.12 | -0.13 | -0.05 | 0.96 | -0.49 | 0.88 | .70 |
| **ASRQ** |  |  |  |  |  |  |  |  |  |
| Acceptance | 25.85 | 3.72 | -0.10 | -0.11 | -0.08 | 0.91 | -0.91 | 0.67 | .87 |
| Emotional support | 25.23 | 3.93 | 0.05 | 0.05 | 0.08 | 0.91 | -1.04 | 1.66 | .87 |
| Intimacy | 23.86 | 3.80 | 0.10 | 0.09 | 0.17 | 0.96 | -0.23 | -0.48 | .85 |
| Instrumental support | 19.84 | 4.70 | 0.19 | 0.18 | 0.18 | 0.97 | 0.27 | -0.34 | .77 |
| Knowledge | 23.95 | 3.80 | 0.13 | 0.11 | 0.27* | 0.92 | -0.81 | 0.57 | .83 |
| **MACH IV** | 17.50 | 4.23 | 0.10 | 0.11 | 0.00 | 0.95 | 0.60 | 0.11 | .72 |
| **STQ** |  |  |  |  |  |  |  |  |  |
| Apathy | 18.63 | 4.21 | -0.07 | -0.06 | -0.05 | 0.96 | 0.43 | -0.52 | .65 |
| Competition | 12.39 | 4.00 | 0.02 | 0.02 | 0.03 | 0.95 | 0.60 | -0.18 | .76 |
| Criticism | 24.46 | 8.07 | 0.00 | -0.02 | 0.06 | 0.93 | 0.96 | 1.40 | .88 |
| Longing | 13.18 | 3.28 | -0.05 | -0.05 | -0.07 | 0.96 | 0.36 | -0.67 | .49 |
| Mutuality | 49.46 | 7.84 | 0.02 | 0.01 | 0.00 | 0.91 | -1.01 | 0.96 | .92 |
| **BIS-11** |  |  |  |  |  |  |  |  |  |
| Motor | 20.66 | 3.20 | -0.04 | -0.03 | -0.10 | 0.97 | -0.20 | -0.09 | .43 |
| Non-planning | 22.14 | 3.52 | -0.10 | -0.08 | -0.18 | 0.98 | -0.19 | -0.11 | .42 |
| Attentional | 16.95 | 3.81 | -0.10 | -0.08 | -0.21 | 0.96 | 0.64 | 0.80 | .52 |

- *Note*: Significance level set to α = 0.05 for individual tests (* = *p*<.05, ** = *p*<.01). The adjusted Bonferroni-corrected α* was 0.017; tests that survived correction are printed bold. ^a^ = Variables normally distributed (*p* < 0.05) as assessed using the Shapiro-Wilk test, while non-marked variables are considered non-normally distributed. BPAQ = Buss-Perry Aggression Questionnaire; RSPQ = Rank Style With Peers Questionnaire; DL = dominant leadership; CB = coalition building; RSA = ruthless self-advancement; DoPL = Dominance, Prestige, and Leadership; HCA = Hypercompetitive Attitude; NEO-FFI = NEO Five-Factor Inventory; O = Openness to Experience; C = Conscientiousness; E = Extraversion; A = Agreeableness; N = Neuroticism; PDCA = Personal Development Competition Attitude; SoPS = Sense of Power Scale; STAXI = State-Trait Anger Expression Inventory; ASRQ = Adult Sibling Relationship Questionnaire; MACH IV = Machiavellianism Scale (selected items); STQ = Sibling Type Questionnaire; BIS-11 = Barratt Impulsiveness Scale 11.
- **Table S4.** Table including descriptives of all dyad-level instrument and subscale scores as well as correlations with dyad mean and difference iTAP scores.

| Variable/ Scale | Descriptives | | Correlation | | | | | |
| --- | --- | --- | --- | --- | --- | --- | --- | --- |
|  |  |  | Dyad mean iTAP score across trials: | | | Dyad difference iTAP score across trials: | | |
|  | *M* | *SD* | All (*n*=65) | Inter. (*n*=60) | Fake (*n*=5) | All (*n*=65) | Inter. (*n*=60) | Fake (*n*=5) |
| **Difference** |  |  |  |  |  |  |  |  |
| **Age** | 2.29 | 1.41 | -0.18 | -0.20 | -0.12 | -0.02 | -0.01 | 0.05 |
| **BPAQ** |  |  |  |  |  |  |  |  |
| Physical Aggression | 4.55 | 3.47 | 0.30 | 0,27 | 0.40* | -0.13 | -0.05 | -0.12 |
| Verbal Aggression^a^ | 2.36 | 1.97 | 0.12 | 0.09 | 0.19 | 0.05 | 0.07 | -0.24 |
| Anger | 4.55 | 4.18 | -0.04 | -0.06 | 0.04 | 0.13 | 0.25 | 0.00 |
| Mistrust^a^ | 3.64 | 3.31 | 0.06 | 0.05 | 0.11 | -0.22 | -0.09 | -0.22 |
| **RSPQ** |  |  |  |  |  |  |  |  |
| DL^a^ | 3.75 | 2.61 | 0.16 | 0.15 | 0.19 | 0.00 | 0.14 | 0.04 |
| CB | 3.04 | 2.85 | 0.15 | 0.15 | 0.16 | -0.03 | 0.04 | -0.24 |
| RSA | 2.82 | 3.58 | -0.02 | -0.02 | 0.02 | 0.22 | 0.28 | 0.25 |
| **DoPL** |  |  |  |  |  |  |  |  |
| Dominance^a^ | 6.69 | 4.45 | -0.17 | -0.18 | -0.03 | -0.24 | -0.19 | -0.11 |
| Prestige^a^ | 5.94 | 4.28 | -0.12 | -0.12 | -0.15 | 0.11 | 0.11 | -0.23 |
| Leadership^a^ | 7.86 | 6.31 | -0.18 | -0.18 | -0.15 | -0.08 | 0.02 | -0.17 |
| **HCA** | 10.55 | 8.03 | 0.04 | 0.06 | 0.04 | -0.04 | -0.04 | 0.09 |
| **NEO-FFI** |  |  |  |  |  |  |  |  |
| O^a^ | 5.26 | 3.83 | -0.32 | -0.31 | -0.31 | -0.13 | -0.02 | -0.09 |
| C^a^ | 3.04 | 2.70 | 0.08 | 0.06 | 0.08 | -0.08 | -0.20 | 0.16 |
| E^a^ | 3.79 | 4.19 | -0.01 | -0.01 | -0.06 | -0.11 | -0.15 | -0.31 |
| A^a^ | 4.46 | 3.14 | 0.25 | 0.25 | 0.24 | -0.22 | -0.22 | -0.25 |
| N^a^ | 5.54 | 4.62 | -0.19 | -0.21 | -0.05 | -0.40* | -0.40* | -0.19 |
| **PDCA** | 8.64 | 5.91 | -0.03 | -0.04 | -0.08 | 0.15 | 0.28 | 0.02 |
| **SoPS** | 0.56 | 0.61 | 0.12 | 0.10 | 0.12 | 0.05 | 0.12 | 0.09 |
| **STAXI** |  |  |  |  |  |  |  |  |
| Trait | 3.86 | 2.81 | -0.02 | -0.04 | 0.04 | 0.03 | 0.17 | -0.22 |
| State | 2.50 | 5.49 | 0.42* | 0.41* | 0.39* | 0.05 | 0.26 | -0.06 |
| In | 5.25 | 3.53 | 0.22 | 0.21 | 0.27 | -0.20 | -0.08 | -0.09 |
| Out | 3.46 | 3.85 | 0.17 | 0.16 | 0.34 | 0.06 | 0.13 | 0.09 |
| Control^a^ | 4.07 | 2.92 | 0.03 | 0.02 | 0.11 | -0.10 | -0.01 | -0.19 |
| **ASRQ** |  |  |  |  |  |  |  |  |
| Acceptance | 3.24 | 2.16 | 0.07 | 0.06 | 0.13 | -0.14 | -0.06 | -0.08 |
| Emotional Support | 2.54 | 1.75 | 0.00 | -0.01 | 0.01 | -0.31 | -0.22 | -0.06 |
| Intimacy | 2.42 | 2.12 | 0.22 | 0.25 | 0.15 | 0.22 | 0.25 | 0.39* |
| Instrumental Support | 3.11 | 2.48 | 0.02 | 0.04 | -0.03 | -0.10 | -0.13 | -0.32 |
| Knowledge | 2.54 | 2.50 | -0.03 | -0.03 | -0.06 | 0.05 | 0.18 | 0.14 |
| **MACH IV** | 3.21 | 2.18 | 0.10 | 0.11 | 0.00 | -0.06 | 0.01 | -0.10 |
| **STQ** |  |  |  |  |  |  |  |  |
| Apathy | 3.61 | 2.88 | -0.09 | -0.09 | -0.03 | -0.01 | -0.10 | -0.16 |
| Competition | 4.36 | 3.50 | -0.06 | -0.06 | 0.04 | -0.18 | -0.23 | -0.10 |
| Criticism | 6.50 | 6.18 | 0.23 | 0.23 | 0.22 | -0.12 | -0.11 | -0.12 |
| Longing | 2.07 | 1.72 | -0.16 | -0.17 | -0.13 | **-0.71**** | **-0.62**** | **-0.67**** |
| Mutuality | 4.71 | 4.24 | 0.12 | 0.12 | 0.22 | 0.05 | 0.16 | 0.07 |
| **BIS-11** |  |  |  |  |  |  |  |  |
| Motor | 2.89 | 2.36 | -0.03 | -0.04 | -0.03 | -0.16 | 0.02 | -0.29 |
| Non-planning | 3.71 | 2.77 | 0.15 | 0.16 | 0.10 | 0.03 | 0.21 | -0.26 |
| Attentional | 4.54 | 3.35 | 0.31 | 0.29 | 0.31 | -0.34 | -0.12 | -0.34 |
| ***Mean*** |  |  |  |  |  |  |  |  |
| **Age** | 23.39 | 3.11 | -0.14 | -0.14 | -0.19 | -0.10 | 0.01 | -0.20 |
| **BPAQ** |  |  |  |  |  |  |  |  |
| Physical Aggression | 15.35 | 4.94 | 0.14 | 0.14 | 0.15 | 0.13 | 0.04 | 0.09 |
| Verbal Aggression^a^ | 13.57 | 2.51 | 0.08 | 0.09 | 0.06 | 0.39* | 0.30 | 0.05 |
| Anger | 14.80 | 4.13 | -0.03 | -0.04 | -0.02 | 0.23 | 0.27 | 0.09 |
| Mistrust^a^ | 18.14 | 3.74 | 0.16 | 0.14 | 0.10 | 0.18 | 0.36 | 0.13 |
| **RSPQ** |  |  |  |  |  |  |  |  |
| DL^a^ | 18.52 | 3.38 | 0.00 | 0.00 | 0.01 | -0.15 | -0.11 | -0.28 |
| CB | 28.63 | 2.76 | -0.07 | -0.06 | 0.01 | -0.13 | -0.01 | -0.10 |
| RSA | 11.27 | 3.18 | 0.15 | 0.15 | 0.16 | 0.08 | 0.19 | 0.07 |
| **DoPL** |  |  |  |  |  |  |  |  |
| Dominance^a^ | 23.73 | 4.59 | 0.20 | 0.20 | 0.17 | 0.33 | 0.30 | 0.13 |
| Prestige^a^ | 42.11 | 6.35 | 0.19 | 0.19 | 0.26 | 0.08 | 0.20 | 0.16 |
| Leadership^a^ | 38.00 | 6.56 | 0.02 | 0.02 | 0.04 | 0.01 | 0.09 | -0.12 |
| **HCA** | 64.26 | 8.07 | 0.10 | 0.09 | 0.09 | -0.10 | 0.09 | -0.04 |
| **NEO-FFI** |  |  |  |  |  |  |  |  |
| O^a^ | 37.40 | 3.86 | -0.22 | -0.23 | -0.27 | 0.18 | 0.15 | -0.01 |
| C^a^ | 38.84 | 2.75 | 0.19 | 0.19 | 0.11 | 0.04 | 0.24 | -0.07 |
| E^a^ | 33.07 | 4.02 | 0.29 | 0.29 | 0.32 | 0.01 | -0.03 | -0.27 |
| A^a^ | 42.45 | 3.24 | 0.06 | 0.07 | 0.07 | -0.10 | -0.06 | -0.14 |
| N^a^ | 33.55 | 3.31 | -0.24 | -0.25 | -0.26 | -0.05 | 0.05 | 0.08 |
| **PDCA** | 52.71 | 8.16 | 0.13 | 0.14 | 0.20 | 0.03 | -0.05 | -0.11 |
| **SoPS** | 4.33 | 0.53 | -0.10 | -0.08 | -0.04 | 0.33 | 0.15 | 0.21 |
| **STAXI** |  |  |  |  |  |  |  |  |
| Trait | 16.75 | 3.34 | 0.13 | 0.13 | 0.12 | 0.30 | 0.41 | 0.10 |
| State | 12.00 | 4.06 | 0.48** | 0.47* | 0.49** | -0.18 | 0.02 | -0.12 |
| In | 16.05 | 3.91 | 0.12 | 0.11 | 0.16 | 0.32 | 0.40* | 0.44* |
| Out | 12.13 | 3.13 | 0.25 | 0.24 | 0.28 | 0.30 | 0.35 | 0.30 |
| Control^a^ | 24.00 | 2.79 | -0.26 | -0.25 | -0.20 | -0.05 | -0.13 | 0.16 |
| **ASRQ** |  |  |  |  |  |  |  |  |
| Acceptance | 25.85 | 3.19 | -0.11 | -0.09 | -0.14 | 0.10 | 0.04 | -0.10 |
| Emotional Support | 25.23 | 3.64 | 0.05 | 0.04 | 0.01 | 0.00 | 0.04 | -0.18 |
| Intimacy | 23.86 | 3.48 | 0.10 | 0.09 | 0.08 | 0.11 | 0.17 | -0.13 |
| Instrumental Support | 19.84 | 4.29 | 0.20 | 0.19 | 0.14 | -0.19 | -0.10 | -0.42* |
| Knowledge | 23.95 | 3.38 | 0.18 | 0.19 | 0.19 | 0.20 | 0.15 | -0.19 |
| **MACH IV** | 17.50 | 3.78 | 0.11 | 0.11 | 0.04 | 0.19 | 0.28 | 0.27 |
| **STQ** |  |  |  |  |  |  |  |  |
| Apathy | 18.63 | 3.55 | -0.13 | -0.14 | -0.12 | 0.14 | 0.11 | 0.12 |
| Competition | 12.39 | 2.88 | 0.05 | 0.04 | 0.12 | 0.18 | 0.12 | 0.18 |
| Criticism | 24.46 | 6.78 | -0.12 | -0.12 | -0.07 | 0.12 | 0.11 | -0.13 |
| Longing | 13.18 | 3.01 | -0.04 | -0.03 | -0.18 | 0.06 | 0.14 | -0.17 |
| Mutuality | 13.18 | 3.01 | 0.02 | 0.03 | -0.07 | 0.17 | 0.14 | -0.04 |
| **BIS-11** |  |  |  |  |  |  |  |  |
| Motor | 20.66 | 2.62 | -0.07 | -0.07 | -0.14 | 0.46* | 0.37 | 0.16 |
| Non-planning | 22.14 | 2.67 | -0.22 | -0.21 | -0.28 | 0.06 | -0.04 | 0.03 |
| Attentional | 16.95 | 2.58 | -0.15 | -0.16 | -0.28 | -0.01 | 0.07 | 0.02 |

*Note*: Significance level set to α = 0.05 for individual tests (* = *p*<.05, ** = *p*<.01). The adjusted Bonferroni-corrected α* was 0.008; tests that survived correction are printed bold. ^a^ = Variables normally distributed (*p* < 0.05) as assessed using the Shapiro-Wilk test, while non-marked variables are considered non-normally distributed.

**Table S5**. Table including descriptives, distribution information and internal consistency of all instruments and subscales as well as correlation with individual mean iTAP scores.

| Variable/ Scale | Descriptives | | Shapiro-Wilks | Distribution | |
| --- | --- | --- | --- | --- | --- |
|  | *M* | *SD* | W | Sk | Ku |
| **Subject** |  |  |  |  |  |
| TAP all | 33.99 | 29.15 | 0.90 | 0.55 | -0.96 |
| TAP interactive | 33.79 | 29.52 | 0.90 | 0.56 | -0.98 |
| TAP fake | 36.29 | 27.88 | 0.91 | 0.48 | -0.45 |
| TAP Zero | 25.95 | 26.03 | 0.80 | 0.46 | -1.56 |
| **Dyad** |  |  |  |  |  |
| Mean TAP all | 33.99 | 29.12 | 0.90 | 0.59 | -0.97 |
| Mean TAP interactive | 33.79 | 29.48 | 0.90 | 0.59 | -1.01 |
| Mean TAP fake^a^ | 36.29 | 26.22 | 0.94 | 0.53 | -0.43 |
| Diff TAP all | 5.59 | 6.31 | 0.75 | 2.39 | 7.04 |
| Diff TAP interactive | 5.45 | 6.64 | 0.75 | 2.24 | 5.80 |
| Diff TAP fake | 15.86 | 12.47 | 0.93 | 0.46 | -0.21 |

*Note*: ^a^ = Variables normally distributed (*p* < 0.05) as assessed using the Shapiro-Wilk test, while non-marked variables are considered non-normally distributed.

## **GEE results**

**Table S6**. Summary of GEE analysis results and post-hoc tests of dyad mean aggression score across blocks and gender.

|  |  | **Post-hoc Tests** | |
| --- | --- | --- | --- |
| **Parameter** | **Estimate (*p*-value)** | **Comparison** | **Difference (*p*-value)** |
| Block | 30.78(< .001)** | Block 1 vs. Block 2 | -1.62 (0.319) |
|  |  | Block 1 vs. Block 3 | −9.52 (< .001)*** |
|  |  | Block 1 vs. Block 4 | −17.46 (< .001)*** |
|  |  | Block 1 vs. Block 5 | −8.70 (0.002)** |
|  |  | Block 2 vs. Block 3 | −7.89 (0.005)** |
|  |  | Block 2 vs. Block 4 | −15.83 (< .001)*** |
|  |  | Block 2 vs. Block 5 | −7.08 (0.008)** |
|  |  | Block 3 vs. Block 4 | −7.94 (< .001)*** |
|  |  | Block 3 vs. Block 5 | 0.82 (0.748) |
|  |  | Block 4 vs. Block 5 | 8.76 (< .001)*** |
| Gender | 0.29(0.588) |  |  |
| Block x Gender | 4.75(0.314) |  |  |

*Note.* * *p* < .05, ** *p* < .01, *** *p* < .001. The significance level for all comparisons has been adjusted to α* = 0.005 after Bonferroni correction.

**Table S7**. Summary of GEE analysis results and post-hoc tests of dyad difference aggression score across blocks and gender.

|  |  | **Post-hoc Tests** | |
| --- | --- | --- | --- |
| **Parameter** | **Estimate (*p*-value)** | **Comparison** | **Difference (*p*-value)** |
| Block | 35.99(< .001)** | Block 1 vs. Block 2 | 3.01 (0.159) |
|  |  | Block 1 vs. Block 3 | −7.62 (< .001)*** |
|  |  | Block 1 vs. Block 4 | −1.60 (0.389) |
|  |  | Block 1 vs. Block 5 | −1.41 (0.334) |
|  |  | Block 2 vs. Block 3 | −10.70 (< .001)*** |
|  |  | Block 2 vs. Block 4 | −4.68 (0.011)* |
|  |  | Block 2 vs. Block 5 | −1.67 (0.166) |
|  |  | Block 3 vs. Block 4 | 6.02 (0.009)** |
|  |  | Block 3 vs. Block 5 | 9.03 (< .001)*** |
|  |  | Block 4 vs. Block 5 | 3.01 (0.159) |
| Gender | 0.06(0.808) |  |  |
| Block x Gender | 6.93(0.139) |  |  |

*Note.* * *p* < .05, ** *p* < .01, *** *p* < .001. The significance level for all comparisons has been adjusted to α* = 0.005 after Bonferroni correction.

- 1. **Cluster Comparison**

**Table S8**. Mean and standard deviation of demographic (upper row) and iTAP scores (lower row) separately for each cluster with respective test. Score description can be found in Table S2.

|  | **Cluster 1**  (*n* = 10) | **Cluster 2**  (*n* = 12) | **Cluster 3**  (*n* = 6) | **Test-**  **statistic(df)** | ***p*-**  **value** |
| --- | --- | --- | --- | --- | --- |
|  | **"Both low aggr."** | **"Mixed aggr."** | **"Both high aggr."** |  |  |
| Male ratio | % | % | % | *χ*^2^(2)^a^ |  |
|  | 60 | 25 | 66.67 | 3.944 | 0.139 |
|  | Mean(SD) | Mean(SD) | Mean(SD) | *H*(2)^b^ |  |
| Age mean | 24.45(2.74) | 21.83(2.63) | 24.75(4.65) | 8.63 | .013 |
| Age difference | 2.3(1.16) | 2.67(1.30) | 1.5(1.87) | 3.17 | .205 |
| M-Inter | 5.49(5.74) | 35.08(15.52) | 78.39(10.52) |  |  |
| M-Fake | 13(11.92) | 37.33(13.80) | 73(18.24) |  |  |
| D-Inter | 3.75(4.74) | 5.19(8.16) | 8.78(5.68) |  |  |
| D-Fake | 15.6(12.64) | 14(8.18) | 20(19.39) | c | |
| M-Zero | 56.75(9.64) | 11.92(12.84) | 2.67(2.38) |  |  |
| D-Zero | 3.7(2.63) | 9(14.03) | 2.33(1.97) |  |  |
| Pre-Post Diff | -7.78(10.33) | -17.17(15.33) | -9.33(20.63) |  |  |

*Note*. a = Chi-square statistic, b = Kruskal-Wallis H-statistic, c = see Table S8 for corresponding tests.

**Table S9**. Results of Kruskal-Wallis tests and post-hoc tests for iTAP scores. For a description of each variable see Table S1.

| **Variable** | **Kruskal-Wallis statistics (*H*)^a^** | ***p*-value** | **Post-Hoc Test** |
| --- | --- | --- | --- |
| D-Fake | 0.32 | 0.850 | - |
| D-Inter | 4.74 | 0.094 | - |
| M-Fake | 18.07 | < .001* | Cluster 1 vs. Cluster 2: *p* < .05 Cluster 1 vs. Cluster 3: p < .001 Cluster 2 vs. Cluster 3: p = .110 |
| M-Inter | 23.15 | < .001* | Cluster 1 vs. Cluster 2: p < .01 Cluster 1 vs. Cluster 3: p < .001 Cluster 2 vs. Cluster 3: p = .084 |
| M-Zero | 19.22 | < .001* | Cluster 1 vs. Cluster 2: p < .01 Cluster 1 vs. Cluster 3: p < .001 Cluster 2 vs. Cluster 3: p = .581 |
| D-Zero | 0.57 | 0.754 | - |
| Pre-Post Diff | 7.96 | < .05 | Cluster 1 vs. Cluster 2: p = .059 Cluster 1 vs. Cluster 3: p = .890 Cluster 2 vs. Cluster 3: p < .05 |

*Note.* ^a^ *df*(2) for Kruskal-Wallis tests. *p* < .05, Bonferroni-corrected alpha level = .007.

**Table S10**. Results of Kruskal-Wallis tests and post-hoc tests of dyad mean and differencescores of instrument subscales and rating scales across clusters.

| **Variable** | **Kruskal-Wallis statistics (*H*)^a^** | ***p*-value** | **Post-Hoc Test** |
| --- | --- | --- | --- |
| **Subscales** |  |  |  |
| BPAQ(Physical Aggression)_Mean_ | 3.43 | 0.180 | - |
| STAXI(State Anger)_Mean_ | 2.94 | 0.230 | - |
| DoPL(Dominance)_Mean_ | 3.66 | 0.160 | - |
| STQ(Competition)_Mean_ | 2.196 | 0.334 | - |
| STQ(Mutuality)_Mean_ | 2.21 | 0.331 | - |
| BPAQ(Physical Aggression)_Diff_ | 3.43 | 0.180 | - |
| STAXI(State Anger)_Diff_ | 3.70 | 0.157 | - |
| DoPL(Dominance)_Diff_ | 2.00 | 0.369 | - |
| STQ(Competition)_Diff_ | 1.146 | 0.564 | - |
| STQ(Mutuality)_Diff_ | 4.967 | 0.083 | - |
| **Ratings** |  |  |  |
| WIN1_Mean_ | 13.34 | 0.001* | Cluster 1 vs. Cluster 2: p = .007 Cluster 1 vs. Cluster 3: p = .005 Cluster 2 vs. Cluster 3: p > .999 |
| LOS1_Mean_ | 8.58 | 0.014* | Cluster 1 vs. Cluster 2: p = .016  Cluster 1 vs. Cluster 3: p =.120  Cluster 2 vs. Cluster 3: p > .999 |
| MOT_Mean_ | 5.25 | 0.07* | - |
| TAP1_Mean_ | 1.97 | 0.373 | - |
| FAIR_Mean_ | 7.80 | 0.020* | Cluster 1 vs. Cluster 2: p = .007  Cluster 1 vs. Cluster 3: p = .005  Cluster 2 vs. Cluster 3: p > .999 |
| TAP7_Mean_ | 4.78 | 0.091 | - |
| R1W_Diff_ | 9.89 | 0.007* | Cluster 1 vs. Cluster 2: p = .021  Cluster 1 vs. Cluster 3: p = .023  Cluster 2 vs. Cluster 3: p > .999 |
| R1L_Diff_ | 7.82 | 0.020* | - |
| MOT_Diff_ | 7.38 | 0.025* | Cluster 1 vs. Cluster 2: p = .523  Cluster 1 vs. Cluster 3: p =.414  Cluster 2 vs. Cluster 3: p = .021 |
| TAP1_Diff_ | 7.00 | 0.030* | - |
| FAIR_Diff_ | 1.19 | 0.551* | - |
| TAP7_Diff_ | 7.14 | 0.028* | Cluster 1 vs. Cluster 2: p = .016  Cluster 1 vs. Cluster 3: p = .033  Cluster 2 vs. Cluster 3: p = .894 |

*Note.* * *p* < .05 ^a^ *df*(2) for Kruskal-Wallis tests. BPAQ = Buss-Perry Aggression Questionnaire; STAXI = State-Trait Anger Expression Inventory; DoPL = Dominance, Prestige, and Leadership; STQ = Sibling Type Questionnaire.
